# Integrated analysis of comprehensive metabolomics and network pharmacology to reveal the mechanisms of *abelmoschus manihot* (L.) medik. in the treatment of cisplatin-induced chronic kidney disease

**DOI:** 10.3389/fphar.2022.1064498

**Published:** 2022-11-18

**Authors:** Jian-Cheng Liao, Chang-Yin Li, Feng-Meng Teng, Jian-Chen, Jiang-Yi Yu, Wen-Zheng Ju, Jian-Dong Zou

**Affiliations:** ^1^ Department of Clinical Pharmacology, Jiangsu Province Hospital of Chinese Medicine, Affiliated Hospital of Nanjing University of Chinese Medicine, Nanjing, China; ^2^ Department of Laboratory Medicine, Jiangsu Province Hospital of Chinese Medicine, Affiliated Hospital of Nanjing University of Chinese Medicine, Nanjing, China; ^3^ Department of Endocrinology, Jiangsu Province Hospital of Chinese Medicine, Affiliated Hospital of Nanjing University of Chinese Medicine, Nanjing, China

**Keywords:** Abelmoschus manihot, chronic kidney disease, metabolomics, network pharmacology, cisplatin

## Abstract

**Background:** Abelmoschus manihot (L.) Medik (“Huangkui” in Chinese, HK) has been widely used for the treatment of kidney diseases. Nephrotoxicity is the side effect of cisplatin (CDDP), which greatly limits its clinical application. Therefore, CDDP could be used to establish the chronic kidney disease (CKD) model. However, the protective effects of HK on CDDP-induced CKD have not been investigated.

**Purpose:** To explore the protective effect and underlying mechanisms of HK on multiple low-dose CDDP-induced CKD in rats by the integrated analysis of serum, kidney, and urine metabolomics and network pharmacology.

**Methods:** The CKD model was induced by multiple low-dose CDDP. Body weight, organ index, serum biochemical, and kidney histology were examined to evaluate the effect of HK. Serum, kidney, and urine were collected and profiled by HILIC/RPLC-Q-TOF/MS-based metabolomics. Potential biomarkers (PBs) were screened according to the criteria of VIP >1, *p* < 0.01, and FC > 2, and then identified or assigned. The pathway analysis and PBs enrichment were conducted by MetaboAnalyst and ChemRICH. Furthermore, network pharmacology was adopted to dig out the active components and targets. Finally, the results from metabolomics and network pharmacology were integrated to confirm each other.

**Results:** HK could recover the CDDP-induced abnormal pharmacological and metabolic profile changes. A total of 187 PBs were screened and identified from the serum, kidney, and urine metabolomics. Pathway analysis showed that multiple metabolic pathways, mainly related to amino acid and lipid metabolisms, were involved in the nephroprotective effect of HK, and especially, HK could significantly alleviate the disorder of tryptophan metabolism pathway in serum, kidney, and urine. Meanwhile, network pharmacology analysis revealed that 5 components in HK and 4 key genes could be responsible for the nephroprotection of HK, which also indicated that the metabolism of tryptophan played an important role in HK against CKD.

**Conclusion:** HK has a nephroprotection on CDDP-induced CKD, mainly by restoring the dysregulation of tryptophan metabolism. Integrated analysis of serum, kidney, and urine metabolomics and network pharmacology was a powerful method for exploring pharmacological mechanisms and screening active components and targets of traditional Chinese medicine.

## 1 Introduction

Chronic kidney disease (CKD) is usually defined as a decline in renal function with a glomerular filtration rate of less than 60 ml/min per 1.72 m^2^ for at least 3 months (Kalantar-Zadeh et al., 2021). A total of 1.2 million people died of CKD in 2017, and 1.4 million cardiovascular disease-related deaths and 25.3 million cardiovascular disease disability-adjusted life years were attributed to kidney injury (Bikbov et al., 2020). Unfortunately, CKD cannot be cured, and existing treatments are limited to chronic dialysis or kidney transplantation (Khattri et al., 2021). At present, serum creatinine (Crea) is a biomarker commonly used to diagnose CKD. However, the concentration of Crea is not changed until kidney function failure has occurred (Wang et al., 2019). Therefore, there is an urgent need to find novel treatments and biomarkers to improve the efficiency of the prevention, treatment, and diagnosis of CKD.

Cisplatin (CDDP) has been widely used to treat many types of cancer, including breast, lung, ovarian, brain, and bladder cancers (Lee et al., 2020). Nephrotoxicity is the main adverse effect of CDDP. Thirty percent of patients treated with CDDP experienced acute kidney injury (AKI) with high mortality. Moreover, patients who have recovered completely from AKI are at a high risk of developing chronic kidney disease (CKD) (Holditch et al., 2019). Therefore, consecutive and multiple injections of CDDP were used to establish the CKD model in several studies (Al Za’abi et al., 2021; Estrela et al., 2021).

Lately, the flowers of *Abelmoschus manihot* (L.) Medik. (pronounced “Huangkui” in Chinese, HK) have been proven to have various biological activities, such as anti-inflammatory and anti-oxidant. The alcohol extraction of HK was first reported in 1995 could improve kidney function and reduce proteinuria of diabetic kidney disease (DKD) (Yu et al., 1995). In 1999, Huangkui Capsule (HKC), the marketed preparation of total flavones in HK, was approved by the Food and Drug Administration for CKD treatment. Since 2015, HK has been listed for the treatment of kidney disease by the Pharmacopoeia of the People’s Republic of China. Several clinical studies have proved the safety and effectiveness of HKC on different chronic kidney diseases, including IgA nephropathy (Li P. et al., 2017), diabetic kidney disease (Zhao et al., 2022), and primary glomerular disease (Zhang et al., 2014). Previous studies have shown that HKC could effectively reduce cisplatin-induced neutrophil gelatinase-associated lipoprotein elevation, indicating that HKC may also have a protective effect on cisplatin-induced kidney injury (Hu et al., 2020). However, the underlying mechanisms of nephroprotection of HK in CDDP-induced CKD have not been reported.

Given the complexity of disease and treatment mechanisms, as an emerging bioanalytical method, metabolomics provides a powerful means to understand disease states and elucidate an overview of physiological and metabolic mechanisms. Liquid chromatography-quadrupole-time of flight mass spectrometry (LC-Q-TOF/MS) is one of the most common analytical techniques used in metabolomics, it can suggest the molecular structure and concentration of metabolites at the same time. Therefore, several studies have applied LC-Q-TOF/MS to understand metabolite changes in serum, urine, and kidney (Chao et al., 2019; Qi et al., 2019). However, metabolites are the final product of biological processes, metabolomics could only reflect the information on terminal changes (Li et al., 2021). It has limited in digging out upstream proteins and genes. Network pharmacology has advantages in exploring the direct interaction between drugs and targets. Therefore, the integration of metabolomics and network pharmacology could reveal the relationship between metabolites and targets, and fully explain the potential active components of the drug and the underlying mechanism of nephroprotection (Wang et al., 2020).

In this study, multiple low-dose administration of CDDP was used to establish the CKD model of rats. The overview of physiological and metabolic mechanisms of HK against CKD was obtained based on serum, kidney, and urine reverse phase liquid chromatography (RPLC)-MS and urine hydrophilic interaction chromatography (HILIC)-MS metabolomics, and active ingredients and targets were screened by network pharmacology. Our results demonstrated that HK could correct the disordered metabolism of serum, kidney, and urine in CKD rats. HK may be a potential drug against CDDP-induced kidney injury.

## 2 Materials and methods

### 2.1 Reagents and materials

Methanol and acetonitrile were of HPLC grade (Merck, United States). MS grade formic acid (lot BCBK9295V, purity: ∼98%) and ammonium formate (lot BCBK2322V, purity: ≥ 99%) were obtained from Fluka-Sigma-Aldrich Chemie (Steinheim, Switzerland). The assay kits for Crea and urea nitrogen (Urea) were purchased from Beckman Coulter Commercial Enterprise (China) Co., Ltd. Ultrapure water was obtained in the laboratory by using the Milli-Q water purification system (Millipore, United States). APCI positive (PN. 4460131, lot A4328) and negative (PN. 4460134, lot A4287) calibration solutions for the AB SCIEX Triple TOF™ system were obtained from the AB Sciex Pte. Ltd., United States CDDP injection (lot 2WA2A1307047B, 20 mg per bottle) was purchased from Qilu Pharmaceutical Co., Ltd. (Hainan, China).

### 2.2 HK extract preparation and identification

HK extract was prepared by the Preparation Department of Jiangsu Provincial Hospital of Traditional Chinese Medicine as follows: HK was first extracted by reflux with 60% ethanol, and the ethanol extract was concentrated to remove alcohol under reduced pressure to obtain the water extract. Then, the water extract was extracted with ethyl acetate, and the ethyl acetate extract was collected and concentrated under reduced pressure on a rotary evaporator, and the resulting material was dried and crushed to obtain the HK extract in powder form, the content of which was > 60% in terms of total flavonoids. The major constituents in HK extract have been identified based on LC-Q-TOF/MS analysis in our previous study (Gao et al., 2020). Briefly, a total of 49 flavones were identified by comparing the retention times and fragment ions of standards and the published literatures, and their corresponding overlaid extract ion chromatograms with compound names were shown in Supplementary Figure S1.

### 2.3 Animal experiment and sample collection

Twenty-four male SD rats (220 g ± 20 g) were obtained from the Shanghai Jiesijie Experimental Animals Co., Ltd. The rats were maintained under standard laboratory conditions with the 12 h light/dark cycle at controlled temperature (22°C–24°C) and humidity (45%–55%), and with free access to food and water. All animal experiments were performed following the guidelines and principles of the Animal Care and Use Committee of the Nanjing University of Chinese Medicine (License No. 2022 DW-31–01).

After being acclimated for seven days, all rats were randomly divided into three groups: normal control rats (Control group), CDDP model rats (CDDP group), and HK-treated model rats (CDDP + HK group). CDDP was administered by intraperitoneal injection to model rats on day 1, 3, 7, 11, and 14, with the daily dosage of 2.5 mg/kg, while control rats received their vehicle in the same manner and volume (0.5 ml/100 g). Meanwhile, HK was administrated to HK-treated model rats from day 1, with a daily dosage of 300 mg/kg (equivalent to the largest clinically recommended daily dose) for 16 consecutive days. On day 18, the urine sample of each rat was collected over a 24 h period, and its volume was accordingly recorded. Then the fresh urine samples were immediately stored at −80°C before analysis. On day 20, all the fasted rats were anesthetized and the blood was collected. Followed by centrifugation (1,900 g, 10 min, 4°C), the serum samples were obtained, divided, and then stored at −80°C until analysis. The liver, kidney, spleen, and thymus were removed and washed immediately with precooled normal saline, and then were weighted for organ index calculation. The left kidney was fixed with 10% neutral-buffered formalin for histopathological examination, while the right kidney was stored at −80°C for metabolomics analysis.

### 2.4 General pharmacological assessment

Rat body weight was monitored from day 1 to day 20 in the whole animal experiment. The organ index was calculated by organ weight/body weight. The serum concentrations of Urea and Crea, two major kidney function indicators, were measured using the Beckman Coulter AU5800 Clinical Chemistry Analyser (Beckman Coulter, United States). Left kidney sections (4 μm) were stained with hematoxylin and eosin and visualized by light microscopy at 200 × magnification for structural evaluation. The data were processed by GraphPad Prism 8 and presented as mean ± SD. The comparison between groups was analyzed using the one-way ANOVA, *p* < 0.05 was considered statistically significant.

### 2.5 Sample preparation for metabolomics study

For serum, 200 μl of the thawed sample was deproteinized with 800 μl of acetonitrile: methanol (1:1, *v/v*) precooled to 4°C, the following procedure is the same way as urine. For kidney, each right kidney was accurately weighed and was then soaked in the precooled acetonitrile: methanol (1:1, *v/v*) solution in the ratio of 1:1 (g:ml). The mixture was vortexed for 30 s, sonicated for 30 min, homogenized by tissue homogenizer, and then vortexed for 30 s again. The suspension (50 μl) was transferred and diluted with 50 μl of water. The mixture was vortexed for 30 s and then centrifuged at 12,000 *g* for 5 min at 4°C. Finally, 5 μl of the supernatant was injected into the LC-Q-TOF/MS system. For urine, an aliquot of 200 μl of the thawed sample was diluted with 800 μl of water: methanol (3:5, *v/v*) precooled at 4°C. The mixture was vortexed for 30 s and then centrifuged at 12,000 *g* for 5 min at 4°C. The supernatant (5 μl) was injected into the HILIC/RPLC-Q-TOF/MS system. For every type of biological sample, 20 μl of the supernatant from each sample was mixed to prepare the corresponding pooled quality control (QC) sample.

### 2.6 Instrumental analysis

For serum and kidney samples, chromatographic separation was performed using a 1,200 HPLC system (Agilent, United States) equipped with an Agilent poroshell 120 SB-C18 column (3.0 mm × 100 mm, 2.7 μm). The column temperature was maintained at 35°C, and the autosampler was set at 8°C. The mobile phase A consists of 2 mM ammonium formate and 0.1% formic acid in water, while the mobile phase B was acetonitrile: methanol (1:1, *v/v*) containing 0.1% formic acid. Gradient elution at a constant flow rate of 0.3 ml/min was employed as follows: 0 min–0.5 min, 30% B; 0.5 min–2 min, 30%–80% B; 2 min–7 min, 80%–90% B; 7 min–15 min, 90%–100% B; 15 min–17 min, 100% B; 17 min–17.1 min, 100%–30% B; 17.1 min–23 min, 30% B.

Both RPLC and HILIC separation was employed for urine analysis. Urine RPLC analysis was similar to that of serum and kidney, only the elution gradient was different, just as follows: 0 min–0.5 min, 5% B; 0.5 min–12 min, 5%–100% B; 12 min–16 min, 16 min–16.1 min, 100%–5% B; 16.1 min–22 min, 5% B; only 1.3 min–16.5 min was switched into MS channel by a Valve Valco 2-Position. For urine HILIC analysis, an Agilent poroshell 120 HILIC column (3.0 mm × 100 mm, 2.7 μm) was utilized, and the mobile phase was changed into 5 mM ammonium formate and 0.05% formic acid in water (A), and acetonitrile (B), and the gradient was adjusted as follows: 0 min–2.5 min, 90% B; 2.5 min–12 min, 90%–70% B; 12 min–20 min, 70%–10% B; 20 min–21 min, 10% B; 21 min–21.2 min, 10%–90% B, 21.2 min–28 min, 90% B.

Mass detection was carried out on a Triple TOF^™^ 5,600 system (AB SCIEX, Foster City, CA) equipped with an electrospray ionization source in both positive and negative ion modes. The detailed parameters used was referring to the methodology described in the previous study (Li C. Y. et al., 2017). Detailed mass spectrum conditions were listed in the Supplementary File.

### 2.7 Quality control

Prior to batch analysis of each type of samples, the corresponding pooled QC sample was analyzed at least five replicates to equilibrate the LC-MS system, and then injected every 10 samples throughout the batch. These QC samples were used to monitor the repeatability and stability of the analytical system.

### 2.8 Data processing and statistical analysis

The raw LC-MS data were preprocessed using MarkerView 1.2.1 (AB SCIEX, Foster City, CA, United States) for peak finding, alignment, filtering, and normalization. The resulting data matrices were composed of sample names, *m/z*-*t*_R_ pairs, and (normalized) ion intensities. The preprocessed data matrices were then imported into SIMCA-P 14.0 (Umetrics, Umea, Sweden) for multivariate statistical analysis, including PCA and the orthogonal partial least-squares discriminant analysis (OPLS-DA). Then the supervised OPLS-DA was performed to further assess the group difference, and 200 times permutation test was utilized to evaluate the predictive ability of the models and their statistical significance. The Q2 values of the OPLS-DA exceeded 0.5, and the differences between R2Y and Q2 were less than 0.4, indicating that the OPLS-DA models were acceptable. The Q2-intercepts in the permutation tests were less than 0.05, demonstrating that models were well validated. The variables responsible for group discrimination could be screened according to the value of variable importance in projection (VIP) derived from the OPLS-DA model. Meanwhile, the two-tailed independent Student’s *t*-test and fold change (FC) analysis were also used to assess the significance of these variables using MarkerView. Variables with VIP > 1, *p* < 0.01, and FC > 2 were considered as significant molecular features (SMFs) between groups.

### 2.9 Metabolite identification

Aiding by software tools including MS-DIAL (version 3.96) (Tsugawa et al., 2015) and PeakView (version 1.2), the screened SMFs were identified or assigned by comparing their accurate MS and MS/MS spectra with that in the integrated MoNA (MassBank of North America) compound library. The MoNA MSP library file used for annotation was generated by combining MS/MS spectra from NIST17 and LipidBLAST (Meissen et al., 2012). If there was no fragment ion information in the databases, the possible structure of SMFs was imported into Peakview, and the theoretical fragment ions were obtained through the fragment pane integrated with the software and matched with the experimental MS/MS spectrum. Only when both the quasi-molecular and the fragment ions were well-matched with the candidate structures, the responding SMFs could be confirmed. Those identified SMFs were finally selected as potential biomarkers (PBs). Data matrices of the PBs were then imported into the Multiple ArrayViewer software package (version 4.6.0) for heatmap generation and hierarchical cluster analysis (HCA).

### 2.10 Metabolic pathway analysis

Before metabolic pathway analysis, the InChiKey of each PB was imported into the web-based Chemical Translation Service (http://cts.fiehn lab. ucdavis.edu/batch) to obtain the corresponding specific number of Kyoto Encyclopedia of Genes and Genomes (KEGG), and PubChem Compound Identifiers (CID). Meanwhile, the simplified molecular-input line-entry system (SMILES) codes for all PBs were obtained from the MSP files or the PubChem Compound Identifier Exchange Service (https://pubchem.ncbi.nlm.nih.gov/idexchange/idexchange.cgi) (Kim et al., 2021). MetaboAnalyst 4.0 (https://www.metaboanalyst.ca/) (Chong et al., 2018) was used for metabolites enrichment analysis, and ChemRICH (http://chemrich.fiehnlab.ucdavis.edu/) (Barupal and Fiehn, 2017) was employed for chemical similarity enrichment calculations of the PBs.

### 2.11 Network pharmacology

The major constituents in HK extract have been identified in the previous report (Gao et al., 2020). Related targets of these compounds were obtained from SwissTargetPrediction (http://www.swisstargetprediction.ch/) (Daina et al., 2019) according to the criteria of probability > 0. The putative target proteins of CKD were obtained from GeneCards (https://www.genecards.org/) (Safran et al., 2021) and DisGeNET (https://www.disgenet.org/) (Pinero et al., 2020) databases. Next, after taking the intersection, the potential targets in HK related to the treatment of CKD were obtained. The component-target network was established using Cytoscape 3.9.1 (Bethesda, MD, United States) (Shannon et al., 2003). According to the average degree to screen the key components and targets. Subsequently, the ClueGO plugin (Bindea et al., 2009) was used to perform the KEGG enrichment analysis.

## 3 Results

### 3.1 Protective effects of HK on CDDP-induced toxicity in rats

**FIGURE 1:**
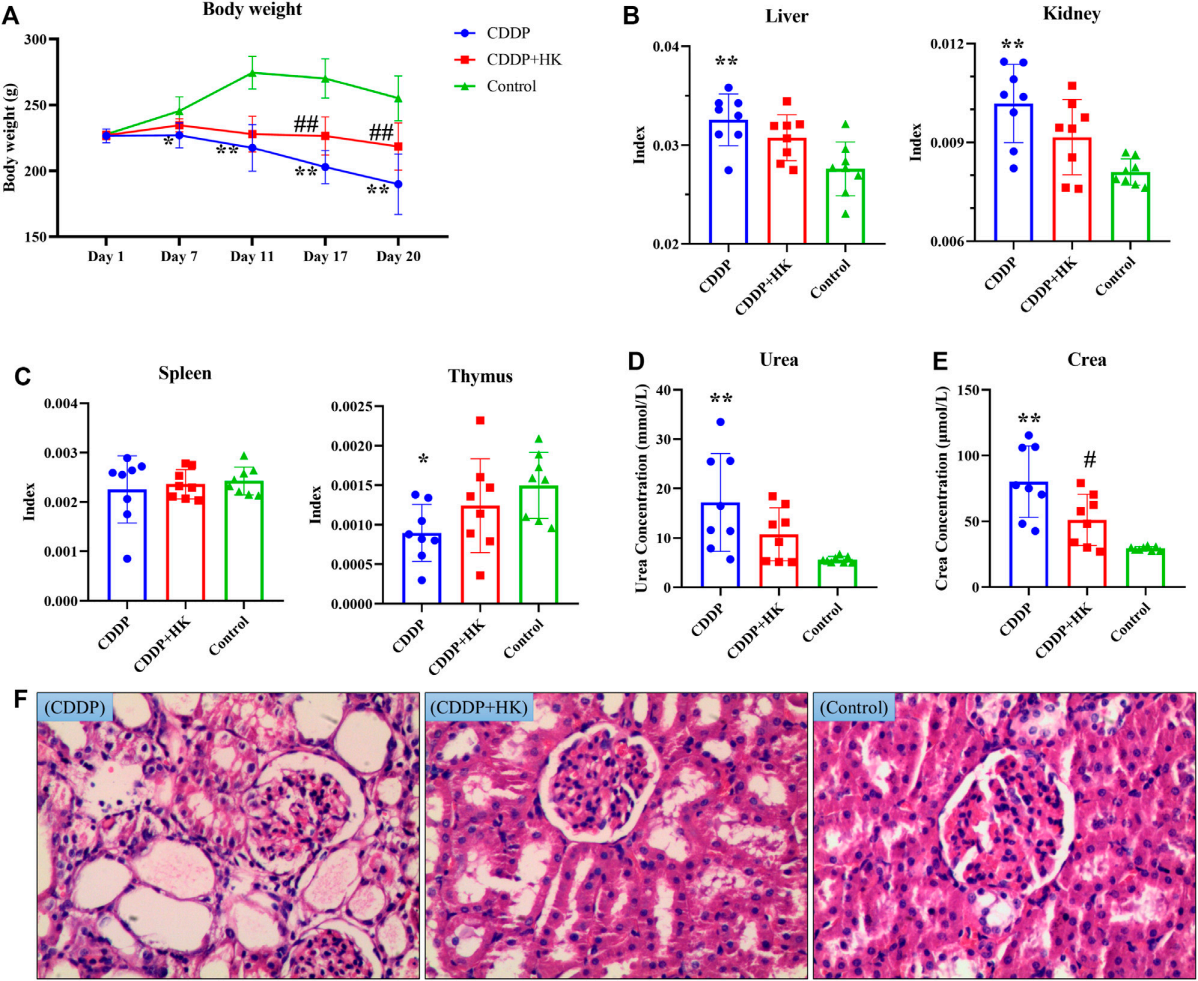
The effects of HK on CDDP-induced toxicity rats. **(A)** The changes in body weight; **(B)** Liver and kidney weight in different groups; **(C)** Spleen and thymus weight in different groups; **(D)** The serum level of Urea; **(E)** The serum level of Crea; **(F)** Representative histopathological section of the kidney. Data were expressed as Mean ± SD in each group. *p < 0.05, **p < 0.01, compared with Control group. ^#^ *p* < 0.05, ^##^ *p* < 0.01, compared with CDDP group.

To determine whether HK possessed protective effects on CDDP-induced toxicity in rats, body weights, organ index, biochemical parameters, and kidney histopathology were measured. As shown in Figure 1A, compared to Control rats, body weight remarkably declined when rats were exposed to CDDP. Liver and kidney indexes were significantly increased on day 20 (Figure 1B), indicating there may be some injury to the liver and kidney, while the thymus index was greatly decreased (Figure 1C), indicating an immune disorder may occur. Serum biochemical parameters and kidney histopathology were examined to further confirm CDDP-induced CKD. As shown in Figures 1D,E, after repeated administration of CDDP, the levels of serum Urea and Crea were significantly upregulated (*p* < 0.01). Under nephroscopy, obvious histological abnormalities such as tubular necrosis, tubule dilatation, and interstitial inflammatory cell infiltration induced by CDDP were observed (Figure 1F). Accordingly, successive oral administration of HK for 16 days could generally recover the above abnormal changes induced by CDDP repeated administration. HK administration could significantly improve the bodyweight loss from day 11, and downregulate the levels of serum Urea and Crea. Besides, to some extent, HK could also alleviate the increase of liver and kidney indexes, the decrease of thymus indexes, and histological abnormality in the kidney. Collectively, all the results from the general pharmacological assessment clearly demonstrated the remarkable toxicity induced by repeated CDDP administration and the definite protection of HK against CDDP-induced toxicity.

### 3.2 Quality control assessment on the metabolomics dataset

**FIGURE 2:**
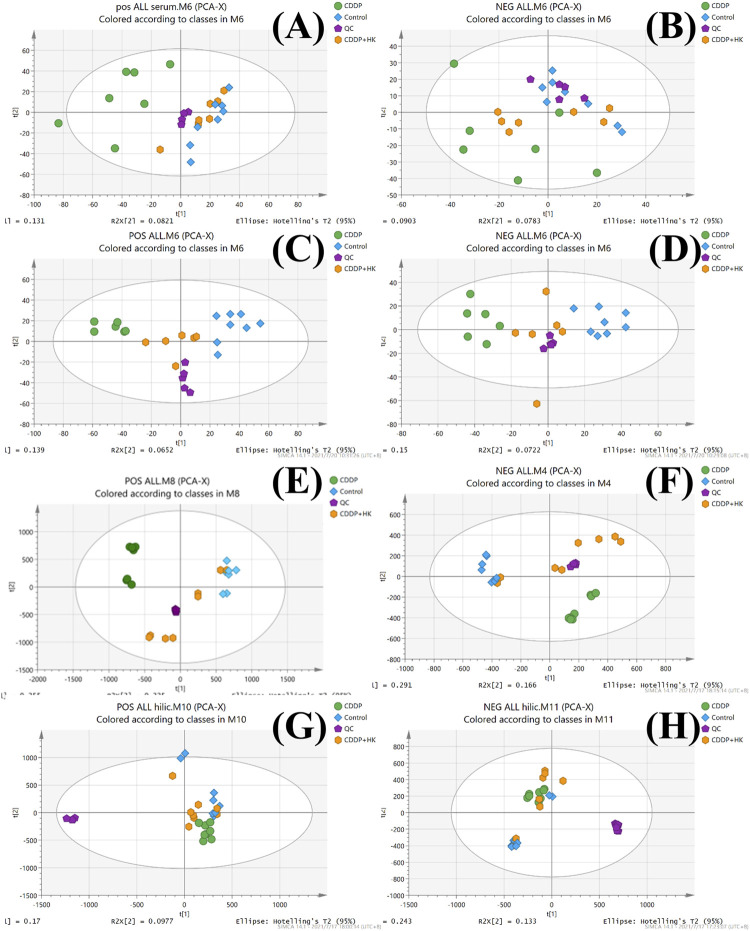
PCA score plots of the Control, CDDP, and CDDP + HK groups. **(A)** Serum samples in positive mode; **(B)** Serum samples in negative mode; **(C)** Kidney samples in positive mode; **(D)** Kidney in negative mode; **(E)** Urine samples for RPLC-Q-TOF/MS analysis in positive mode; **(F)** Urine samples for RPLC-Q-TOF/MS analysis in negative mode; **(G)** Urine samples for HILIC-Q-TOF/MS analysis in positive mode; **(H)** Urine samples for HILIC-Q-TOF/MS analysis in negative mode.

The overlaid LC-MS total ion chromatograms (TICs) from the pooled QC samples of rat serum, kidney, and urine were shown in Supplementary Figure S2. It could be seen that each sample type displayed its corresponding unique metabolite profile in both positive and negative ion modes. For each type of sample, the TICs of all pooled QC samples possessed excellent overlap, indicating that the instrumental system was kept stable during the sample analysis, thereby ensuring that the acquired metabolomics datasets from serum, kidney, and urine were of good quality. Furthermore, as shown in Figure 2, QC samples from each type of metabolomics dataset were generally well clustered in the corresponding PCA score plots, further demonstrating the metabolomics data acquisition was reliable and could be subjected to the following data processing and statistical analysis.

### 3.3 Metabolic profile analysis

**FIGURE 3:**
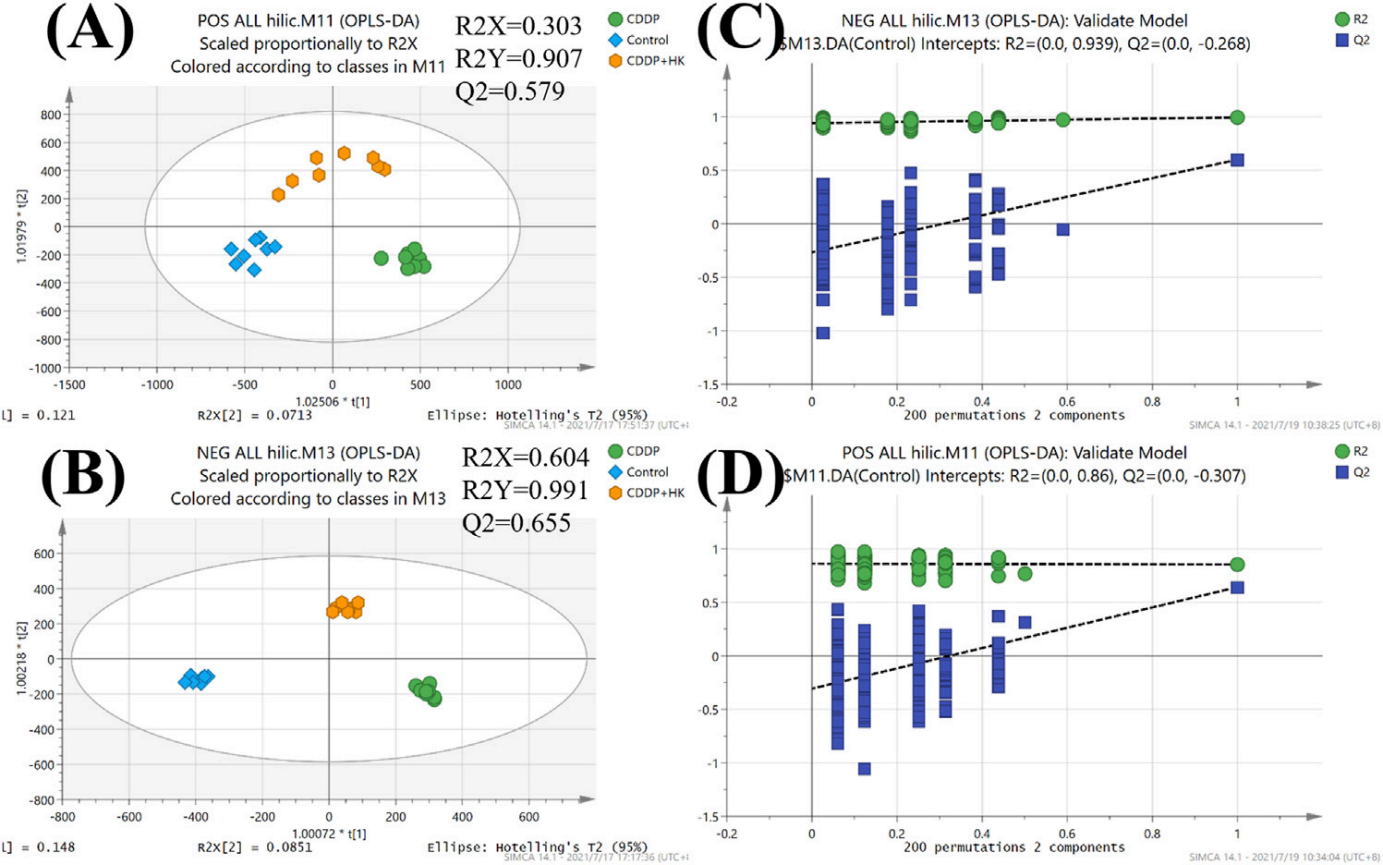
OPLS-DA score plots and permutation tests of urine from Control, CDDP, and CDDP + HK groups for HILIC-TOFMS analysis. **(A)** OPLS-DA score plots in positive mode; **(B)** OPLS-DA score plots in negative mode; **(C)** Permutation tests in positive mode; **(D)** Permutation tests in negative mode.

As an unsupervised analysis mode, PCA analysis could provide an overview of the metabolic profiles of all the samples unbiasedly. As shown in Figures 2A–F, a distinct clustering was observed in each group for all three kinds of samples in both positive and negative ion modes in the PCA plots. These results clearly demonstrated that repeated administration of CDDP and HK could remarkably alter the metabolic profiles in rat serum, kidney, and urine. Meanwhile, the CDDP + HK group was basically located in the middle of the CDDP and Control groups, indicating that 16 consecutive days of HK administration could significantly recover CDDP-induced abnormal metabolic alteration in serum, kidney, and urine. However, as shown in Figures 2G,H, the discrimination of urinary metabolic profiles derived from HILIC-Q-TOF/MS analysis was not very remarkable in the PCA score plots, especially in negative ion mode. Certain types of metabolites only detected in HILIC analysis may contribute to this inconsistency. OPLS-DA analysis was used to further explore the difference in the HILIC-related metabolic profiles among groups. As shown in Figure 3, the OPLS-DA models were acceptable, permutation test results indicated that our models were well validated. Furthermore, all HILIC-analysis samples could be closely clustered within groups and well separated among groups, and the CDDP + HK group was just located between the other two groups in OPLS-DA models. The above results fully demonstrated the definite treatment effect of HK in CKD from the perspective of metabolic profiles.

OPLS-DA analysis was further employed to display the discrimination of metabolic profiles between every two groups (i.e., Control vs*.* CDDP, CDDP vs*.* CDDP + HK) and screen the SMFs between groups. As shown in Supplementary Figure S3–S6, the quality of all OPLS-DA score plots was acceptable, and permutation test results indicated that our models were well validated, VIP values from these OPLS-DA models could be used for SMF screening. Samples from different groups presented a discrete state in the corresponding OPLS-DA score plots, indicating a clear metabolic difference between groups.

### 3.4 Serum metabolic pathway analysis for the protection of HK

**FIGURE 4:**
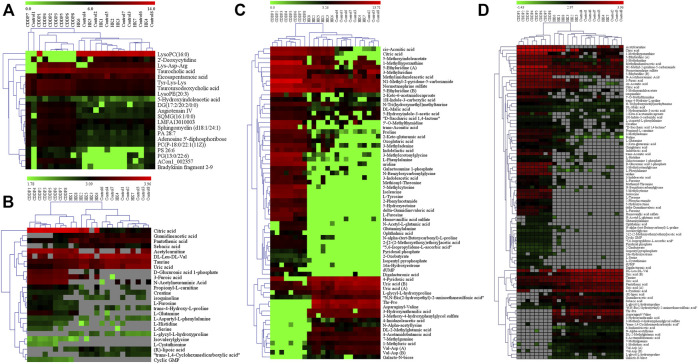
The heatmap and hierarchical cluster analysis of relative contents of potential biomarkers. **(A)** Serum; **(B)** Urine from HILIC-Q-TOF/MS analysis; **(C)** Urine from RPLC-Q-TOF/MS analysis; **(D)** Kidney. The color demonstrated the clear up- or down-regulation of the PBs in 3 groups, while HCA analysis displayed the correlation of the samples (from the sample tree) and the identified PBs (from the gene tree).

**FIGURE 5:**
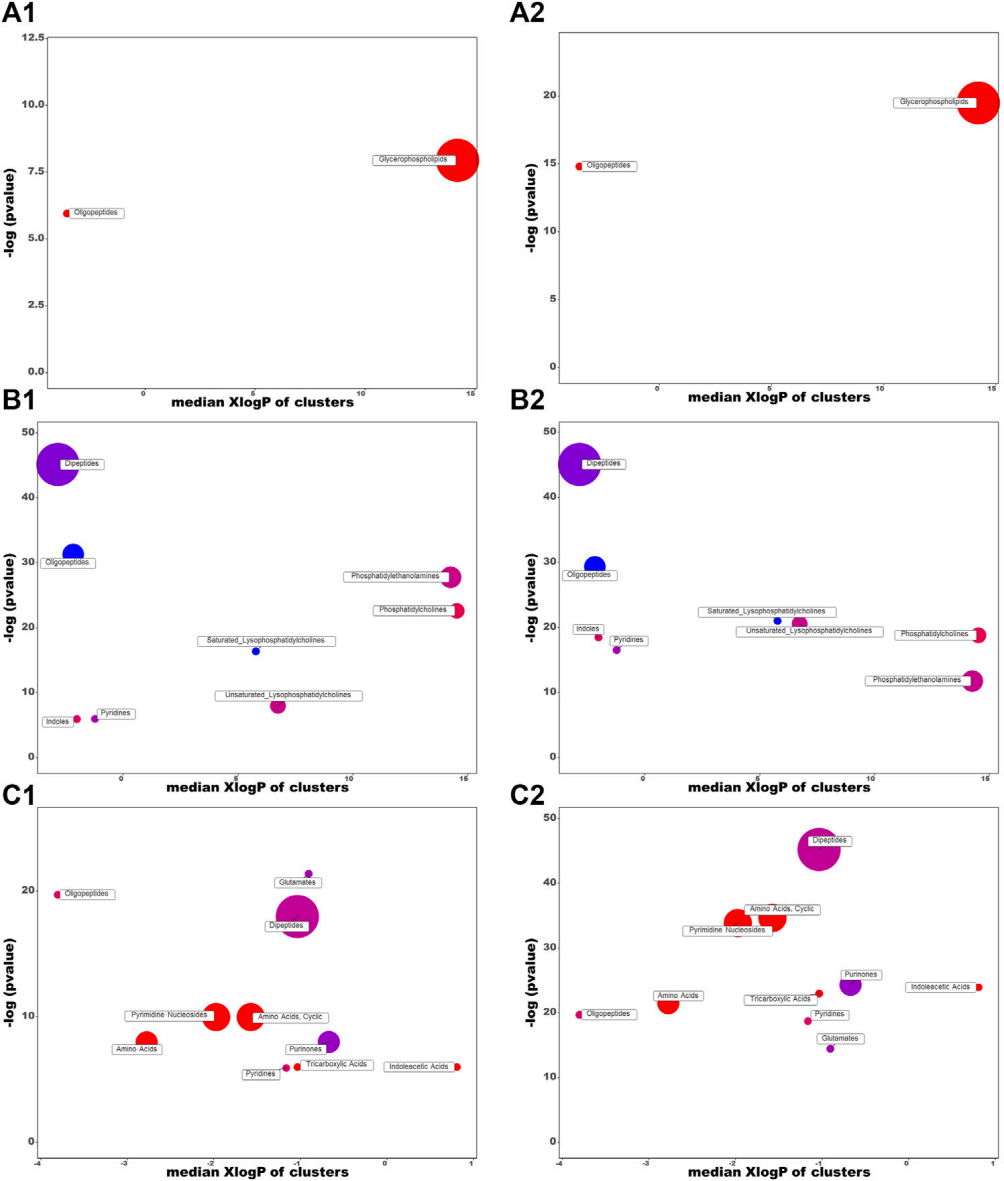
Chemical similarity enrichment analysis of the HK- attenuated PBs in the serum **(A)**, kidney **(B)**, and urine **(C)**. (1) and (2) represent CDDP vs. Control and CDDP vs. CDDP + HK, respectively. Cluster colors give the proportion of increased or decreased compounds (red = increased, blue = decreased) in each cluster. Dots size indicates the number of PBs in each cluster. The top of the *Y*-axis shows the most obvious clusters. Chemical enrichment statistics are calculated by Kolmogorov–Smirnov test.

According to the criteria of VIP > 1, *p* < 0.01, and FC > 2, twenty-one differential metabolites were screened as PBs of HK treatment. The detailed information of these PBs, including *t*_R_, *m/z*, ion type, name, and cluster label from ChemRICH, PubChem CID, as well as the values of *p*, FC, and VIP from group comparison, were summarized in Supplementary Table S1, while the corresponding heatmap and HCA were shown in Figure 4A. The CDDP + HK group was closer to Control than CDDP in the structure of the sample tree, indicating that the HK administration could recover the identified PBs abnormality of CKD. Chemical similarity enrichment calculations were performed to provide the significantly altered clusters in all types of samples using ChemRICH. The significantly enriched altered metabolites clusters mainly were glycerophospholipids and oligopeptides, which levels were increased significantly in the CDDP group compared to Control, HK administration could reverse the upregulation of these PBs induced by CDDP injection (Figure 5A).

**FIGURE 6:**
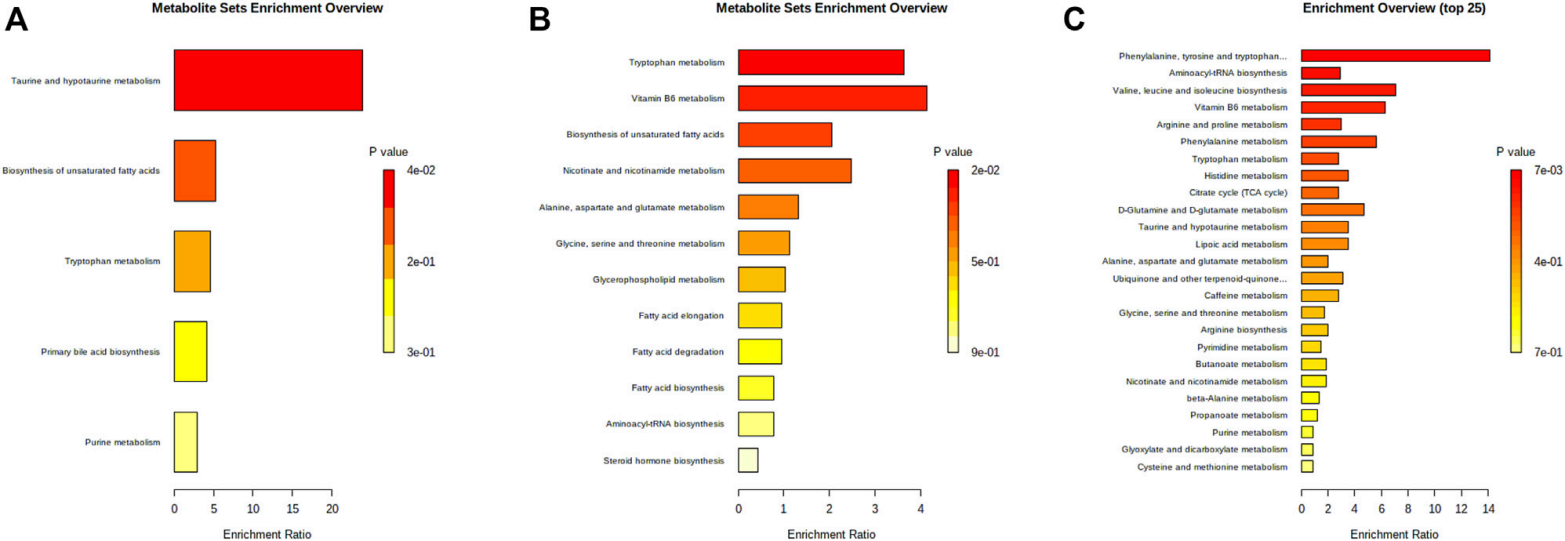
Bar charts of enrichment analysis based on the HK-attenuated PBs in serum **(A)**, kidney **(B)**, and urine**(C)**.

Moreover, PBs enrichment analysis was performed using MetaboAnalyst. It should be noted that only seven out of 21 PBs could be enriched with valid KEGG IDs (most dipeptides and some lipids could not be mapped). There were five dysregulated pathways (Figure 6A) related to the HK nephroprotection effect, including two pathways involved in lipid metabolism (biosynthesis of unsaturated fatty acid and primary bile acid), two pathways associated with amino acid metabolism (tryptophan metabolism, taurine and hypotaurine metabolism), and one pathway related to nucleotide metabolism (purine metabolism). The enriched metabolite set with *p* < 0.1 was the taurine and hypotaurine metabolism, with an increased taurocholic acid in the CDDP group.

### 3.5 Kidney metabolic pathway analysis for the protection of HK

According to the criteria of VIP > 1, *p* < 0.01, and FC > 2, a total of 81 differential metabolites in the kidney were screened as treatment PBs of HK treatment. The detailed information of these PBs, including *t*_R_, *m/z*, ion type, name, cluster label from ChemRICH, PubChem CID, as well as values of *p*, FC, and VIP from group comparison, were summarized in Supplementary Table S2, while the corresponding heatmap and HCA were shown in Figure 4D. The CDDP + HK group was closer to Control than CDDP in the structure of the sample tree, indicating that HK administration could recover the identified PBs abnormality of CKD. These enriched significantly altered metabolites could be roughly divided into eight clusters according to chemical similarity enrichment calculations, including dipeptides, oligopeptides, phosphatidylethanolamines (PE), phosphatidylcholines (PC), saturated lysophosphatidylcholines (LPC), unsaturated LPC, indoles, and pyridines (Figure 5B). In contrast to the CDDP + HK and Control groups, in the CDDP group, oligopeptides, saturated LPC, and a major of dipeptides were reduced; while PC, indoles, and a majority of PE, unsaturated LPC, and pyridines were increased in the kidney.

When analyzing the kidney metabolites, 30 out of the 81 altered metabolites could be mapped with valid KEGG IDs. A total of 12 disrupted pathways were enriched (Figure 6B). There were six disrupted pathways associated with lipid metabolism (including biosynthesis of unsaturated fatty acid (FA), FA and steroid hormone, metabolism of glycerophospholipid, FA elongation and degradation), and four pathways were related to amino acid metabolism (including tryptophan, alanine, aspartate, glutamate, glycine, serine, and threonine metabolism, aminoacyl-tRNA biosynthesis), two pathways were related to the metabolism of cofactors and vitamins (including vitamin B6, nicotinate and nicotinamide metabolism). The enriched metabolite set with *p* < 0.1 was the tryptophan metabolism, with decreased L-tryptophan in the CDDP group, while 5-Hydroxyindoleacetic acid and L-kynurenine were increased.

### 3.6 Urine metabolic pathway analysis for the protection of HK

According to the criteria of VIP > 1, *p* < 0.01, and FC > 2, a total of 68 and 26 identified PBs were selected as treatment PBs of HK treatment, from the urine RPLC-Q-TOF/MS and urine HILIC-Q-TOF/MS, respectively. The detailed information on these PBs was summarized in Supplementary Table S3–S4. The heatmap and HCA (Figures 4B,C) indicate that HK administration could recover the identified PBs abnormality of CKD. Three pairs of isomers with the same change trend among three groups, including uric acid (rPB7 and rPB16), 5-Ethyluridine (rPB5 and rPB18), and Val-Asp (rPB9 and rPB17), were tentatively identified from RPLC urine dataset. Four urine PBs (uric acid, L-glycyl-L-hydroxyproline, L-furosine, citric acid) were detected in both RPLC and HILIC analysis with the same change trend among the three groups, and therefore totally 90 urine PBs were identified. These enriched significantly altered metabolites could be roughly divided into 10 clusters (Figure 5C). Compared to CDDP + HK and Control groups, oligopeptides, amino acids (including cyclic), pyrimidine nucleosides, tricarboxylic acids, and indoleacetic acids clusters of the CDDP group were increased in urine sample; most glutamates, dipeptides and pyridines were increased; and a majority of purinones were decreased in urine.

When analyzing the urine metabolites, 43 out of the 90 altered metabolites could be mapped with valid KEGG IDs. There were 29 disrupted pathways were enriched, and the top 25 most significant pathways were shown in Figure 6C. Most disrupted pathways were associated with the metabolism of amino acids, lipids, carbohydrates, nucleotides, cofactors, and vitamins. Seven significant pathways were enriched with *p* < 0.1. The most significant one was phenylalanine, tyrosine, and tryptophan biosynthesis (*p* < 0.01), with increased L-phenylalanine and L-tyrosine in the CDDP group. The others were aminoacyl-tRNA biosynthesis, valine, leucine and isoleucine biosynthesis, vitamin B6 metabolism, phenylalanine metabolism, and tryptophan metabolism.

### 3.7 Comprehensive analysis of serum, kidney, and urine metabolomics

**FIGURE 7:**
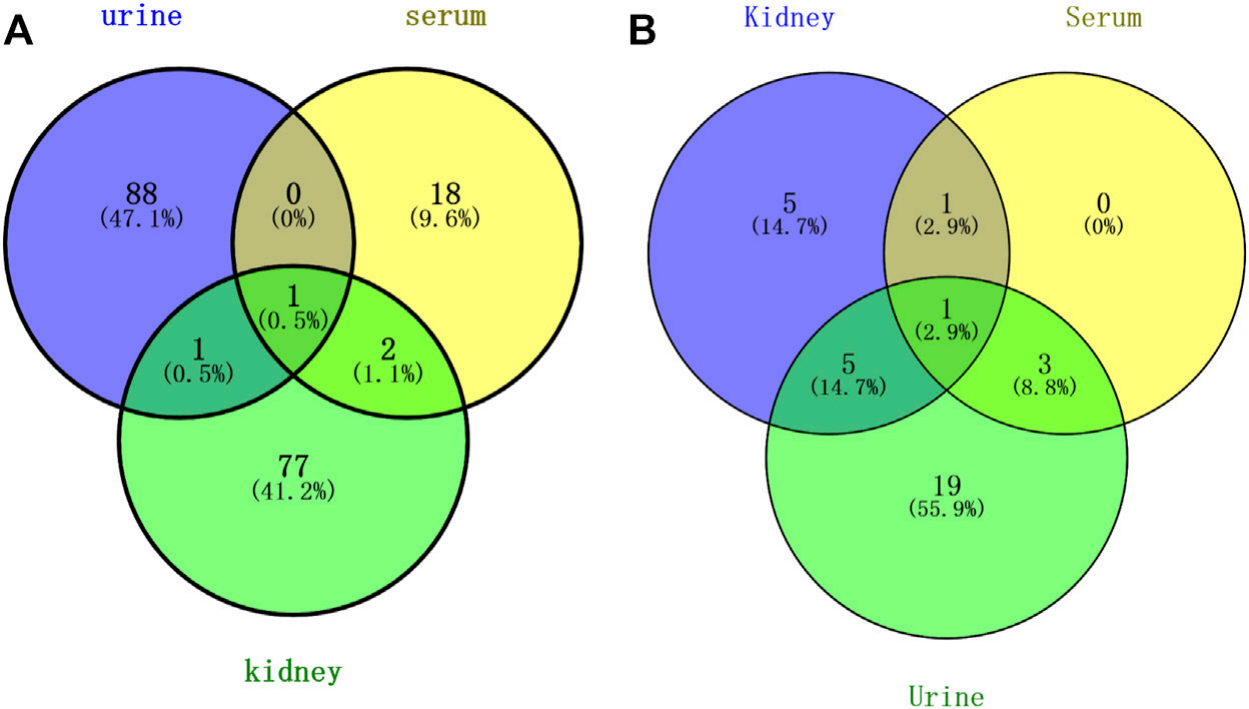
Venn analysis of potential biomarkers **(A)** and pathways **(B)** in rat urine, serum, and kidney.

Above all, a total of 187 PBs were screened to represent the treatment effect of HK on CKD in the three types of samples. Among them, 90, 21, and 81 PBs were selected from the urine, serum, and kidney dataset, respectively. As shown in Figure 7A, a total of three PBs [5-hydroxyindoleacetic acid, eicosapentaenoic acid, and LPC (16:0)] were common in serum and kidney, two PBs (5-hydroxyindoleacetic acid, ophthalmic acid) were common in urine and kidney, and only one PB (5-hydroxyindoleacetic acid) was common in serum, urine, and kidney. These PBs were roughly divided into 7 clusters, including amino acid, peptide (oligopeptide, dipeptide), lipid (glycerophospholipid, PE, PC, saturated LPC, unsaturated LPC), indole, tricarboxylic acid, pyridine, and pyrimidine. A comparison was made against serum, kidney, and urine metabolomics. ChemRICH results showed that oligopeptides were increased in both serum and urine after injection of CDDP, while decreasing in the kidney. The trend of indole and purine PBs in urine was partly similar to that in the kidney.

**FIGURE 8:**
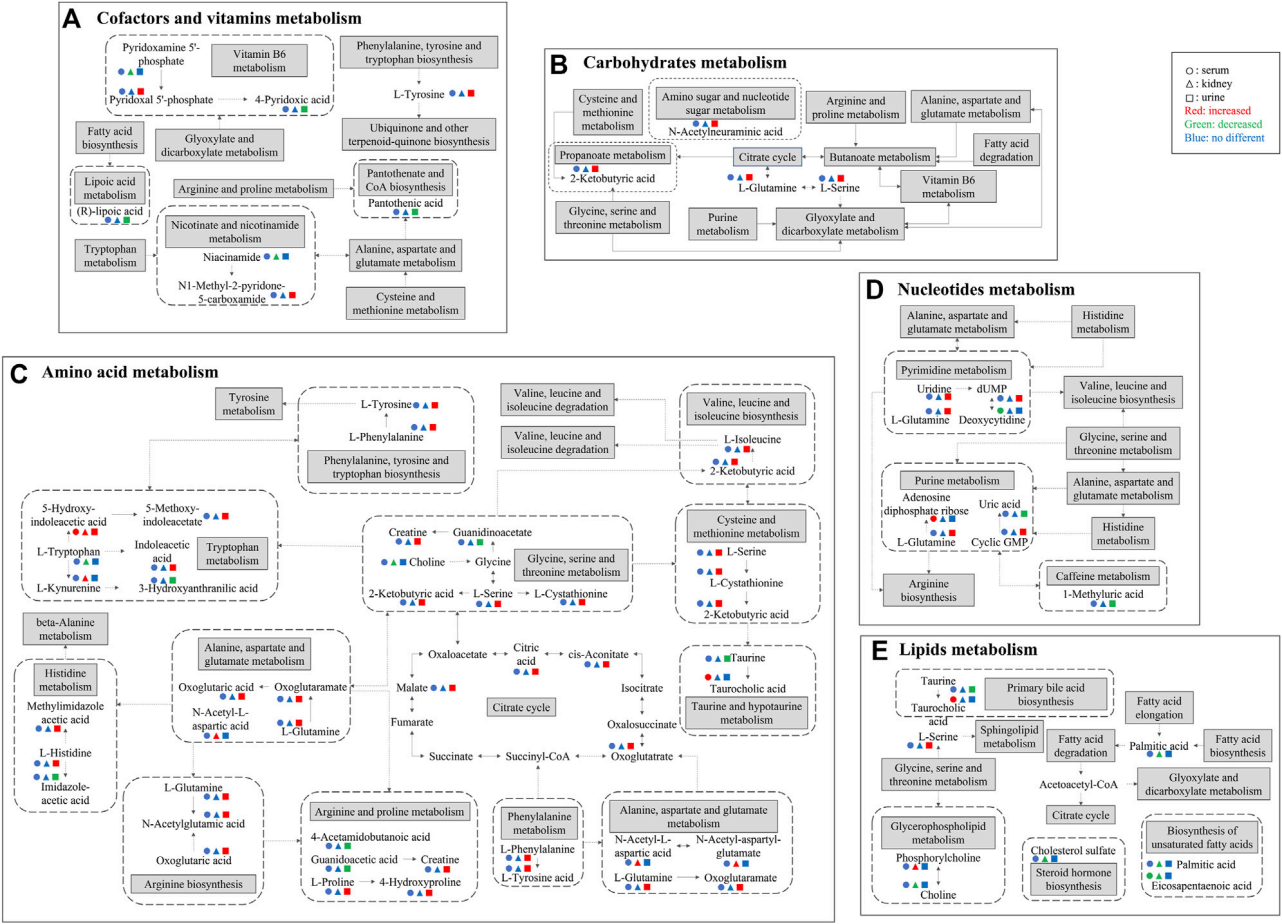
Major differential metabolites and metabolic pathways interrupted by CDDP and regulated by HK. **(A)** Cofactors and vitamins metabolism; **(B)** Carbohydrates metabolism; **(C)** Amino acid metabolism; **(D)** Nucleotides metabolism; **(E)** Lipids metabolism. Solid arrows indicate direct effects and dotted arrows indicate indirect effects. The attenuated markers in serum, kidney, and urine are represented by circle, triangle, and rectangle respectively. Compared to CDDP + HK and Control groups, increased, decreased, and “no different” levels are presented in red, green, and blue, respectively.

As shown in Figure 7B, a total of two pathways (tryptophan metabolism and unsaturated FA biosynthesis) were common in kidney and serum, and six pathways (tryptophan metabolism, vitamin B6 metabolism, alanine, aspartate and glutamate metabolism, aminoacyl tRNA biosynthesis, and glycine, serine and threonine metabolism) were common in kidney and urine, four pathways (tryptophan metabolism, primary bile acid biosynthesis, purine metabolism, and taurine and hypotaurine metabolism) were common in serum and urine, and only tryptophan metabolism was the common pathway in serum, kidney, and urine. The mainly disrupted pathways in HK treating CKD were displayed in Figure 8.

### 3.8 Network pharmacology analysis

**FIGURE 9:**
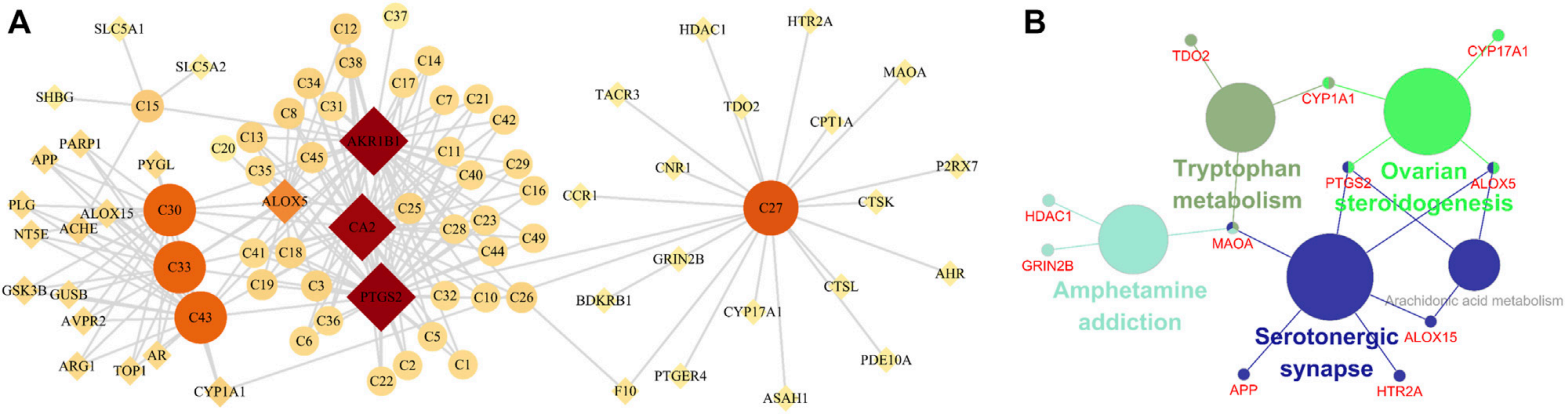
Network pharmacology analysis. **(A)** The component-target network. Circle nodes indicate active components in HK, diamond nodes represent the targets, and the size and color of the node are determined by the degree value. **(B)** KEGG enrichment analysis.

Network pharmacology was used to screen the potential active ingredients and targets, and to further explore the mechanisms of HK against CKD. A total of 49 flavones of HK extract have been identified in the previous report, among them, 42 ingredients may be related to the treatment of CKD, details were listed in Supplementary Table S5. A total of 103 related targets of HK were predicted. Disease targets were obtained in GeneCards and DisGeNET, and 3,422 targets were retrieved. Taking the intersection of HK- and disease-related targets, a total of 40 targets were potential targets directly involved in HK in the treatment of CKD (Supplementary Table S6). And then, the component-target network was constructed by Cytoscape. As shown in Figure 9A, according to degree > 4.8, five components (isopongaflavone, gossypetin, myricetin, quercetin, and liquiritin) and four targets (AKR1B1, PTGS2, CA2, and ALOX5) were key and may play an important role in treating CKD.

Subsequently, to interpret the metabolic mechanisms of potential targets, the ClueGO plugin was used to perform a KEGG enrichment analysis. As shown in Figure 9B, the KEGG analysis indicated tryptophan metabolism, ovarian steroidogenesis, amphetamine addiction, serotonergic synapse, and arachidonic acid metabolism play important roles in HK against CKD.

Integrating the results of the pathway analysis of comprehensive metabolomics and the KEGG analysis of network pharmacology revealed that the tryptophan metabolism overlapped.

## 4 Discussion

Traditional Chinese medicine (TCM) has the characteristics of slow action, so the CKD model is more suitable to explore the nephroprotection mechanisms of HK. In the present study, the body weight of the CDDP + HK group had not shown significant change compared to the CDDP group until day 11. In addition, the pathway analysis showed that multiple pathways are involved in CKD. The TCM has a variety of components, which could act on multiple targets at the same time and play a synergistic effect. Therefore, TCM may have more advantages in nephroprotection.

Metabolomics is an effective tool for studying the overall changes of metabolites *in vivo*. In the present study, the mechanisms of HK on CKD rats were investigated by comprehensive untargeted metabolomics analysis, in which three types of biological samples including serum, kidney, and urine were analyzed. There are a lot of non-polar metabolites in serum and kidney, while a lot of polar metabolites are in the urine. Therefore, HILIC-Q-TOF/MS was used to supply the metabolomics of polar compounds in urine.

Serum metabolomics study could deliver information on metabolism in the whole body, which is similar to that HK against CKD through multi-components and multi-targets. Kidney metabolomics studies could directly discover the mechanisms of CKD. Urine is the end of metabolism and has an intimate connection with renal function. So comprehensive metabolomics of serum, kidney, and urine was necessary to dig out the full mechanisms of HK in treating CKD.

Amino acids were increased in serum and urine, while decreased in the kidney. Amino acids are not only cellular signaling molecules, but also regulators of gene expression and protein phosphorylation cascades. In addition to being components of proteins and peptides, some amino acids are involved in key metabolic pathways necessary for the regulation of homeostasis, growth, reproduction, and immunity (Wu et al., 2020). In most mammals, 99% of the amino acids are reabsorbed in kidney tubules after filtration, and only a very small number of amino acids are excreted into the urine. However, after cisplatin administration, renal tubule reabsorption was decreased, glomerular permeability was increased, and renal amino acid content was sharply decreased in the kidney, while increased in the urine and serum (Zhang et al., 2017), which is consistent with our results, indicated that renal tubule and glomerular are main damage regions in CKD.

The amino acids associated with phenylalanine, tyrosine and tryptophan biosynthesis were increased in the urine. Phenylalanine is an unstable essential amino acid, and the accumulation of phenylalanine metabolites could affect symbiotic bacteria *in vivo*, and may lead to changes in energy metabolism, inflammatory response, intestinal permeability, and systemic immunity (Dodd et al., 2017). Phenylalanine could be converted to tyrosine by phenylalanine carboxylase, tyrosine is involved in energy metabolism and immunity (Ashe et al., 2019). Interestingly, the HK administration could correct the level of these metabolites.

The tryptophan metabolism was enriched in both integrated metabolomics and network pharmacology. Tryptophan is an important aromatic amino acid involved in various physiological processes, such as cell growth and coordination of environmental and dietary responses, mainly metabolized in the gastrointestinal tract (Agus et al., 2018; Kaluzna-Czaplinska et al., 2019). Tryptophan-related metabolites (5-hydroxy-indoleacetic acid and L-kynurenine) were elevated in the kidney, while tryptophan was decreased. At the same time, in agreement with kidney metabolomics results, indoles (5-hydroxy-indoleacetic acid, 5-methoxy-indoleacetate, indoleacetic acid) were increased in the urine after CDDP administration. These changes were reversed by HK, which suggests that gut microbiota plays a key role in the process of HK attenuating CKD. The changed tryptophan and its indole metabolites may indicate that CDDP-induced kidney injury is associated with the regulation of oxidative stress, inflammation, and immune responses (Song et al., 2021). Recent studies have shown that tryptophan and its metabolites are essential biomarkers in both chronic and acute kidney disease (Tan et al., 2021). For example, the accumulation of indoxyl sulfate, one of the metabolites of tryptophan, is usually a sign of kidney function insufficiency (Devlin et al., 2016). Therefore, the level of tryptophan metabolites may be used as biomarkers for the diagnosis of CDDP-CKD. It remains unclear that how exactly the tryptophan metabolites and their corresponding targets are involved in the therapeutical effect of HK against CKD, further targeted metabolomics studies and molecular mechanism investigations are still needed to address this issue.

In this study, glycerophospholipids (including PC, PE, and LPC) had significant changes in serum and kidney after CDDP administration, and HK could correct these changes. Glycerophospholipids are the main part of cell membranes. Disruption of glycerophospholipid metabolism could lead to changes in cell membrane composition, leading to a variety of pathological processes (Qu et al., 2020). It has been suggested that the inflammatory response activated by NF-κB is involved in glycerophospholipid metabolism, which may be one of the mechanisms of kidney injury induced by cisplatin (Yuan et al., 2017). PC could be deacylated at its *sn*-2 position by the enzymatic action of phospholipase A_2_ (PLA2) to release a fatty acid and *sn*-1 LPC is generated (Afshinnia et al., 2017). Furthermore, LPC is subsequently reacylated to form PC by the acyl-CoA-dependent enzyme, LPC acyltransferase (LPCAT). LPCAT could utilize both saturated and unsaturated LPC substrates at the same rate, to synthesize PC (Gopalam et al., 2021). PE is a precursor of PC and a substrate for post-translational modification, which affects the topological structure of the membrane and promotes the membrane fusion of cells and organelles (Afshinnia et al., 2019; Gao et al., 2021). In the present study, PCs were increased while LPCs and PE were partly decreased in the kidney after CDDP administration (Figure 5B). The variation of PC, PE, and LPC content in the kidney suggested that enzyme activities in the kidney changed after CDDP injection, lead to changed components in the cell membrane and result in the altered permeability of cell membranes. Subsequently, the cell membrane was breakdown, and glycerophospholipids in serum were elevated. This is consistent with the renal tubule injury we observed. Owing to the lipids of the same kind usually share the common KEGG ID (e.g., all PCs corresponding to C00157), MetaboAnalyst built-in pathway enrichment analysis was not very suitable for pathway annotation of lipid metabolites. ChemRICH could partly complement the structure grouping and relationship among the lipid potential biomarkers, but fail to provide the corresponding pathway information. Therefore, new effective tools are still needed to be developed for comprehensive analysis of lipid pathways.

After CDDP injection, tricarboxylic acids (TCA), including cis-aconitic acid, trans-aconitic acid, and citric acid were increased in urine. This change may be also associated with renal tubular cell damage, resulting in reduced reabsorption and increased excretion of TCA. Studies showed that mitochondria could produce reactive oxygen species (ROS) under the stimulation of cisplatin, leading to mitochondrial damage and lipid oxidation, and disordering the TCA cycle and related intermediate metabolite levels. However, changes in these metabolite levels are only non-specific reactions of organ damage, thus cannot be used as reliable biomarkers for cisplatin-induced CKD (Won et al., 2016; Zhang et al., 2016).

Uric acid is involved in oxidative stress. It is speculated that the mechanism of CDDP-induced kidney injury may be involved in the excessive consumption of uric acid, which could release a large number of ROS; this results in increased oxidative stress and further activation of Toll-like receptor signaling pathways, which promote downstream NF-κB protein phosphorylation and lead to inflammatory responses, ultimately leading to kidney injury (Huang et al., 2019). Pyrimidines are widely distributed in mammals and provide materials for many kinds of metabolism in mammals throughout their lives. Promoting cell proliferation is the main function of pyrimidine metabolism. Uridine is the basis of DNA replication and plays an important role in protecting the host from pathogenic microorganisms (Liu et al., 2021). Purines and pyrimidines play key roles in DNA replication and RNA synthesis (Lv et al., 2020). Therefore, the disorder of purine and pyrimidine metabolism could affect cell proliferation, which is consistent with the principle of CDDP therapy for tumors. In this study, purines, including uric acid, 4-pyridoxic acid, 7-methylguanine, and 1-methyl uric acid were decreased in urine administration of CDDP. However, the increased pyrimidines were contrary to previous reports, and the related mechanism needs to be further investigated.

Further, pathway analysis showed that the taurine and hypotaurine metabolism was the most significant change pathway in the serum of CKD rats. The taurine and hypotaurine metabolism is associated with antioxidant and cell growth, and increased levels of metabolites in these pathways in response to CDDP- induced kidney injury may be due to the accumulation of free radicals and increased lipid peroxidation (Qu et al., 2020).

However, metabolomics supports the interaction between PBs and related pathways and has limits in the elucidation of direct action between components and targets. Network pharmacology could quickly screen the metabolites, targets, and pathways related to HK against cisplatin-induced CKD, and further explain the mechanisms of action. The key active targets (AKR1B1, PTGS2, CA2, and ALOX5) of HK were identified according to network pharmacology analysis. AKR1B1 (Aldo-Keto Reductase Family 1 Member B) is an aldose reductase enzyme, that can catalyze the reduction of glucose to sorbitol and is highly expressed in the kidney (Huang et al., 2021). Carbonic anhydrase catalyzes the conversion of CO_2_ to bicarbonate in renal tubules. CA2 (Carbonic Anhydrase 2) is present in the cytoplasm of all acidified renal tubules, which accounts for 95% of renal carbonic anhydrase activity. The inhibition of carbonic anhydrase reduced the bicarbonate reabsorption rate of the proximal tubule by 90%, which explains the increased levels of tricarboxylic acids in urine(Baum et al., 2013). In addition, PTGS2 (Prostaglandin-Endoperoxide Synthase 2) and ALOX5 (Arachidonate 5-Lipoxygenase) both are proinflammatory enzymes, that catalyze the metabolism of arachidonate to prostaglandins and leukotrienes, involved in the inflammatory reaction of CKD (Li et al., 2018; Beltrán-Noboa et al., 2022). It has been reported that PTGS2 is positively correlated with the severity of the inflammatory reaction and then aggravates the damage of renal tubular epithelial cells (Fan et al., 2021). It suggested that inhibiting the expression of PTGS2 can alleviate CKD. Nevertheless, network pharmacology analysis focuses on protein-protein interaction, the direction of the effect and the related epigenetic regulation need to be further studied (Chan et al., 2022).

Moreover, the total flavone of HK has shown prominent anti-inflammatory, antitumor, antioxidant, and nephroprotective effects (Zhang et al., 2019). This may indicate that HK can not only against CKD, but also improve the antitumor effect of CDDP, this needs to be verified by further experiments. According to the network pharmacology analysis results, gossypetin, myricetin, and quercetin were key components, indicating that these compounds were active ingredients of HK in treating CKD. Meanwhile, the main compounds and their content in HK are hyperoside (43.2%), hibifolin (27.1%), isoquercitrin (13.7%), quercetin-3′-*O*-D-glucopyranoside (8.8%), quercetin-3-O-robinobioside (3.8%), myricetin (3.2%), and quercetin (0.2%) (Luan et al., 2020). These compounds contain the unit of active ingredients, fully showing that HK has significant advantages in the treatment of CKD.

## 5 Conclusion

Our research was focused on exploring the effect and mechanism of HK against CKD by integrated analysis of serum, kidney, and urine metabolomics and network pharmacology. The current results indicated that the therapeutic effects of HK on CKD are mainly involved in amino acid metabolism, glycerophospholipid metabolism, TCA cycle, and purine metabolism, indicating the underlying mechanism may be related to the inhibition of oxidative stress and inflammatory response. Tryptophan metabolism was proved to be the major target pathway for the nephroprotection of HK on CDDP-induced CKD by the comprehensive metabolomics analysis of serum, kidney, and urine, as well as the ingredients-based network pharmacology analysis. The present study not only demonstrated that the integrated analysis of metabolomics and network pharmacology was powerful and necessary to elucidate potential mechanisms of HK against CKD but also provides an alternative nephroprotection strategy for CDDP-based cancer therapy.

## Data Availability

The datasets presented in this study can be found in online repositories. The names of the repository/repositories and accession number(s) can be found in the article/Supplementary Material.
