# Supplementary material for: Integrated analysis of comprehensive metabolomics and network pharmacology to reveal the mechanisms of abelmoschus manihot (L.) medik. in the treatment of cisplatin-induced chronic kidney disease
Source: Front Pharmacol. 2022 Nov 18;13:1064498. doi: 10.3389/fphar.2022.1064498 (PMC9715753; doi:10.3389/fphar.2022.1064498)
Supplement: Supplementary file 1 [file DataSheet1.docx]

Integrated analysis of comprehensive metabolomics and network pharmacology to reveal the mechanisms of Abelmoschus Manihot (L.) Medik. in the treatment of cisplatin-induced chronic kidney disease

Jian-Cheng Liao^1^, Chang-Yin Li^1^*, Feng-Meng Teng^2^, Jian-Chen^1^, Jiang-Yi Yu^3^, Wen-Zheng Ju^1^, Jian-Dong Zou^1^

^1^ Department of Clinical Pharmacology, Jiangsu Province Hospital of Chinese Medicine, Affiliated Hospital of Nanjing University of Chinese Medicine, No. 155 Hanzhong Road, Nanjing 210029, China

^2^ Department of Laboratory Medicine, Jiangsu Province Hospital of Chinese Medicine, Affiliated Hospital of Nanjing University of Chinese Medicine, No. 155 Hanzhong Road, Nanjing 210029, China

^3^ Department of Endocrinology, Jiangsu Province Hospital of Chinese Medicine, Affiliated Hospital of Nanjing University of Chinese Medicine, No. 155 Hanzhong Road, Nanjing 210029, China

*Correspondence to: Chang-Yin Li

E-mails: fsyy00612@njucm.edu.cn

Tel.: +86-25-8658-7807

# S1 LC-MS conditions

A 1200 HPLC system (Agilent, USA) equipped with an Agilent Poroshell 120 SB-C18 column (100 mm × 3.0 mm, 2.7 μm) was utilized to separate the HK extract. The column temperature was maintained at 35℃, and the autosampler was set at 8℃. The mobile phase A consists of 5 mM ammonium formate and 0.1% formic acid in water, while the mobile phase B was acetonitrile: water (90:10, *v/v*) containing 5 mM ammonium formate and 0.1% formic acid. Gradient elution at a constant flow rate of 0.3 mL/min was employed as follows: 0-0.5 min, 5% B; 0.5-12 min, 5-100% B; 12-16 min, 100% B; 16-16.1 min, 100-5% B; 16.1-22 min, 5% B; 17-17.1 min, 100-30% B; 17.1-23 min, 30% B.

The Triple TOFTM 5600 (AB SCIEX, Foster City, CA) equipped with electron spray ionization source was used for MS detection, and both positive and negative ion modes were employed. The ion spray voltage floating was set at 5500V and -4500 V for positive and negative ion mode respectively. TOF/MS scan conditions were as follows: TOF mass range was set at m/z 100~1000; accumulation time: 0.25 s; ion source gas 1: 60 psi; ion source gas 2: 60 psi; curtain gas: 35 psi; heater temperature: 550°C; declustering potential: 80V; collision energy 10 eV; The options of IDA, DBS and high sensitivity were chosen. Major IDA switch criteria were as follows: Intensity exceeds 500 cps, exclusion isotope within 4Da, mass tolerance 50 mDa, the maximum number of candidate ions to monitor per cycle 8. For Product Ion scan type, TOF mass range was set at m/z 50~1000, accumulation time was set at 0.100006 s, collision voltage set at 35±15 eV, ion release delay at 67 ms, ion release width at 25 ms, and the other parameters were same with TOFMS scan type. All the operations and acquisitions were controlled by Analyst® TF 1.6 software (AB SCIEX, Foster City, CA).


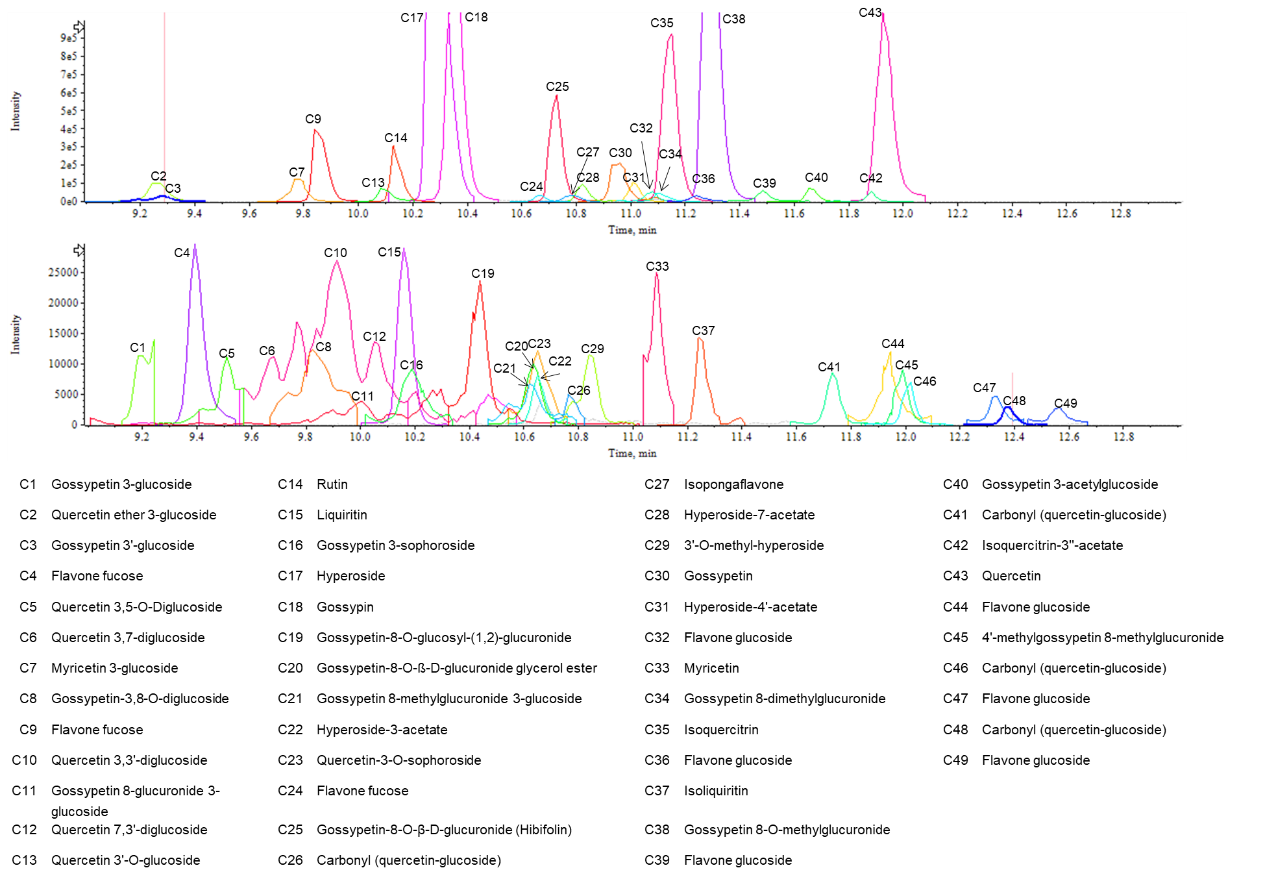


# Fig. S1 The extract ions chromatogram of 49 flavones in HK extract.

**
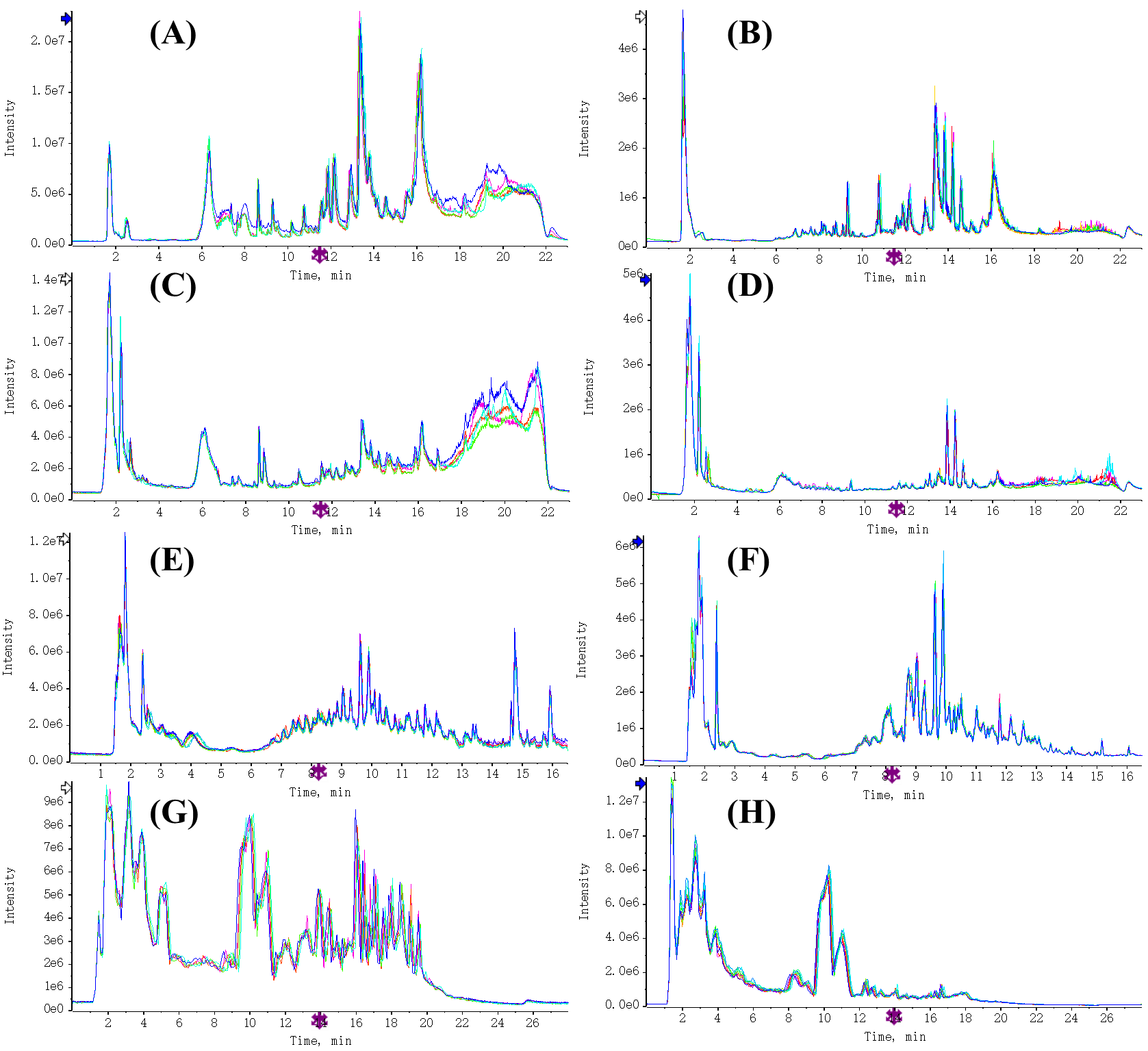
**

# **Fig. S2** The overlaid LC-QTOF-MS/MS or HILIC-QTOF-MS/MS total ion chromatograms from pooled QC samples of rat serum, kidney and urinsamples in both positive and negative ion modes. (A) positive ion mode, serum; (B) negative ion mode, serum; (C) positive ion mode, kidney; (D) negative ion mode, kidney; (E) positive ion mode, urine; (F) negative ion mode, urine; (G) positive ion mode, urine; (H) negative ion mode, urine.

**
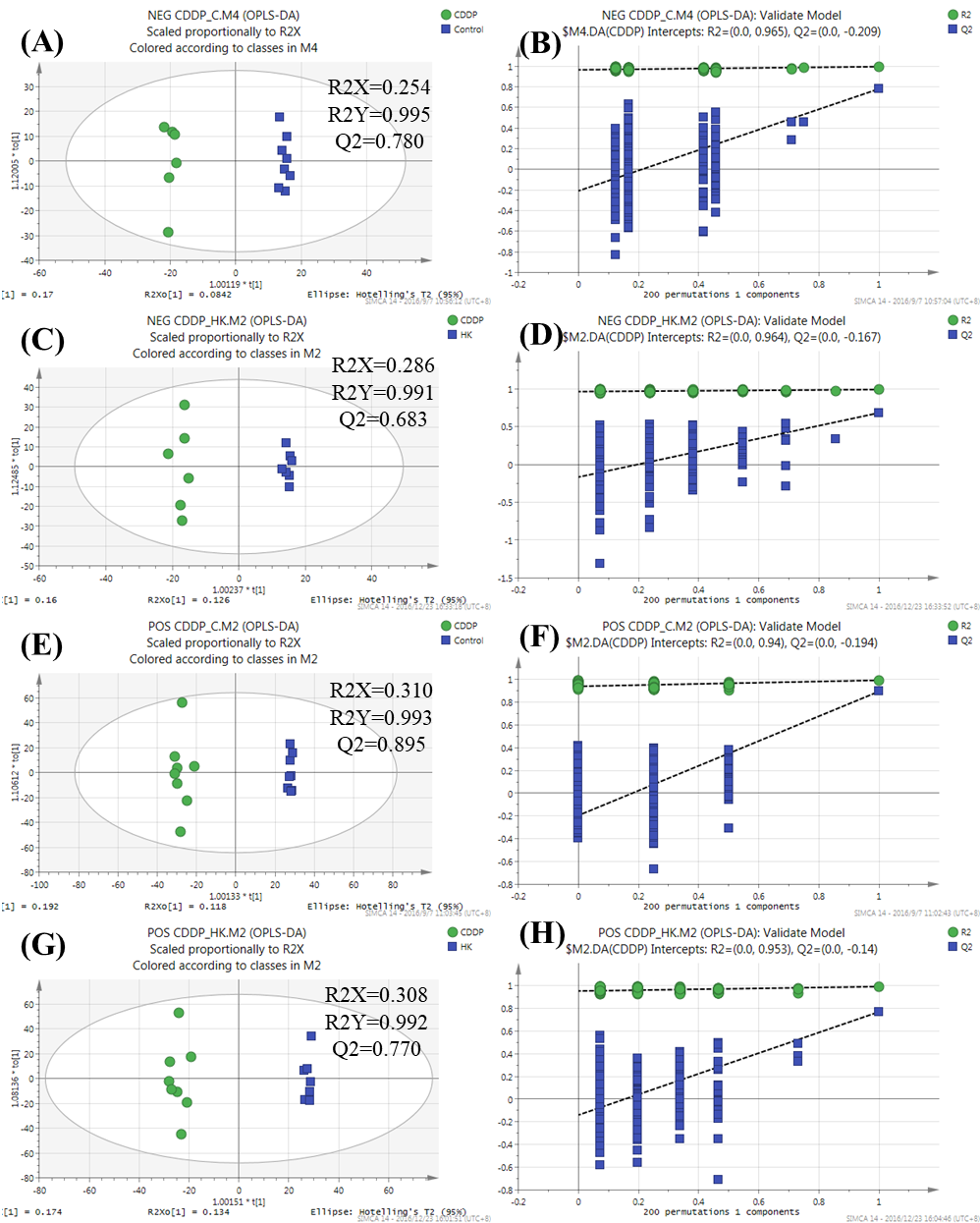
**

# Fig. S3 OPLS-DA score plots and permutation tests of serum LC-TOFMS analysis in negative and positive ion modes. Control *vs.* CDDP groups in negative (A, B) and positive (E, F); Control *vs.* CDDP+HK groups in negative (C, D) and positive (G, H).

**
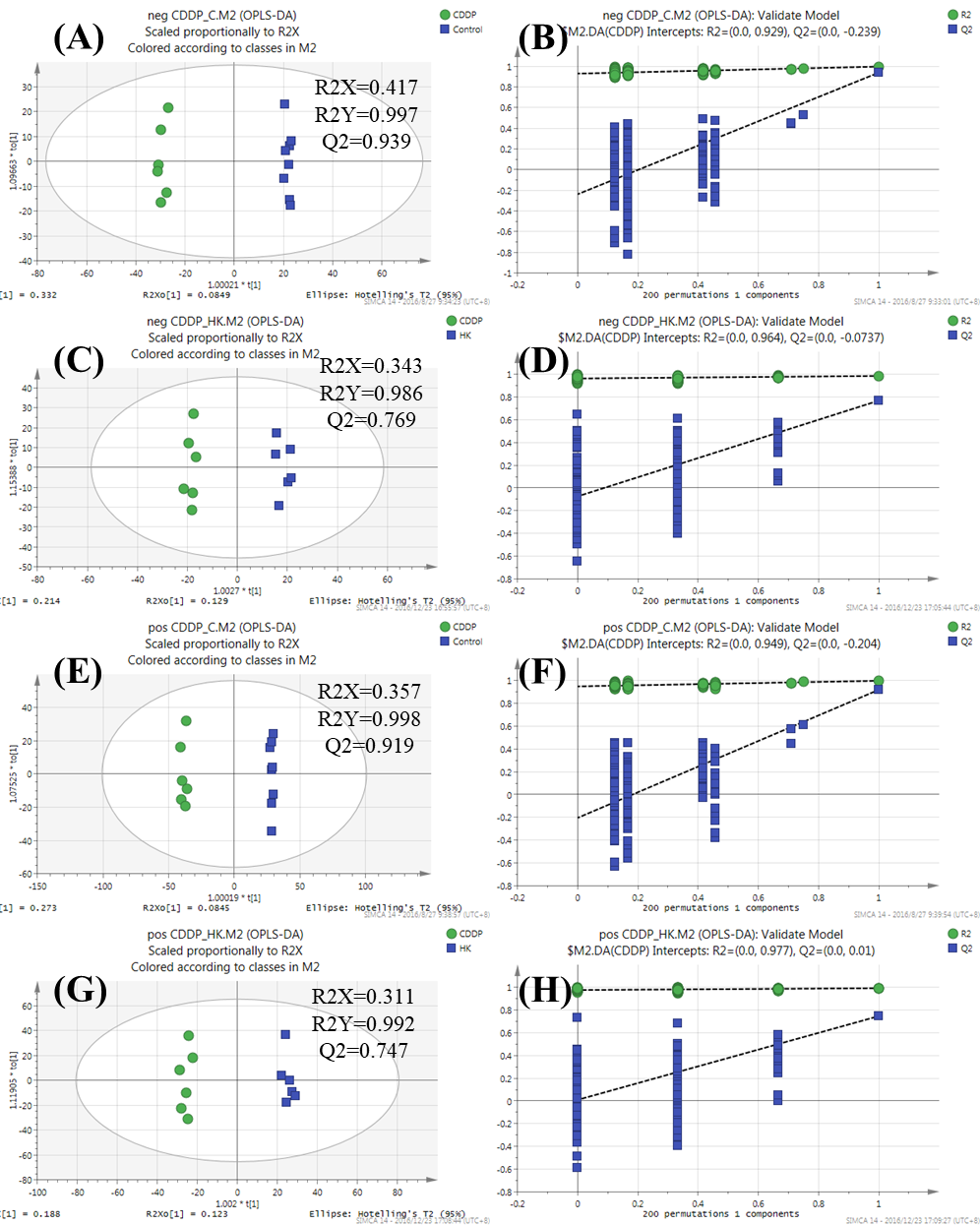
**

# Fig. S4 OPLS-DA score plots and permutation tests of kidney LC-TOFMS analysis in negative and positive ion modes. Control *vs.* CDDP groups in negative (A, B) and positive (E, F); Control *vs.* CDDP+HK groups in negative (C, D) and positive (G, H).

**
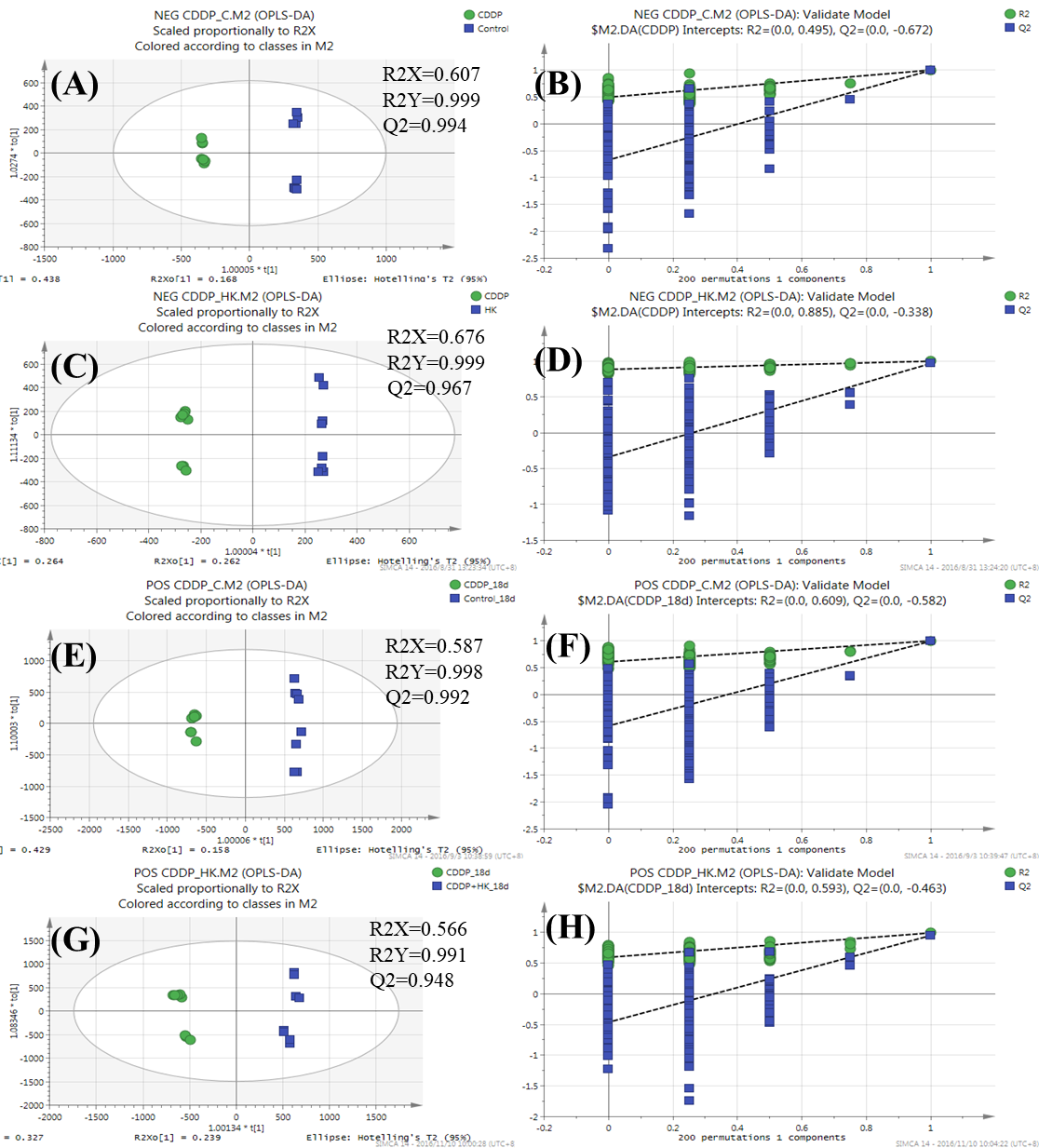
**

# Fig. S5 OPLS-DA score plots and permutation tests of urine LC-TOFMS analysis in negative and positive ion modes. Control *vs.* CDDP groups in negative (A, B) and positive (E, F); Control *vs.* CDDP+HK groups in negative (C, D) and positive (G, H).

**
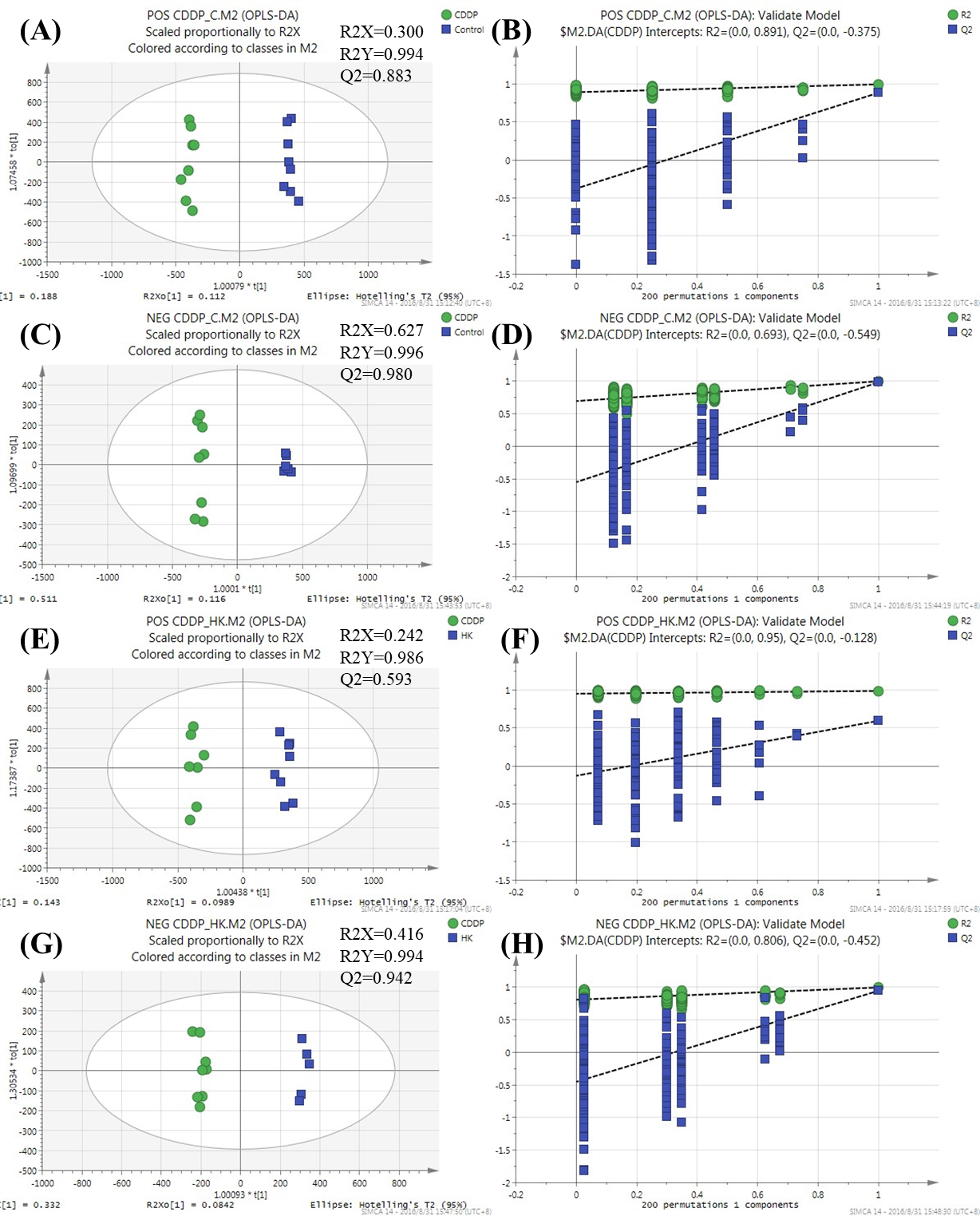
**

# Fig. S6 OPLS-DA score plots and permutation tests of urine HILIC-TOFMS analysis in negative and positive ion modes. Control *vs.* CDDP groups in negative (A, B) and positive (E, F); Control *vs.* CDDP+HK groups in negative (C, D) and positive (G, H).

# Table S1 The list of 21 nephrotoxicity-attenuation PBs of HK in the serum.

| No. | RT (min) | *m/z* (Da) | Ion Type | Formula | Metabolite Name | Cluster Label | PubChem CID | CDDP to Control | | | CDDP to HK | | |
| --- | --- | --- | --- | --- | --- | --- | --- | --- | --- | --- | --- | --- | --- |
|  |  |  |  |  |  |  |  | *p* | FC | VIP | *p* | FC | VIP |
| PB1 | 1.81 | 455.1872 | [2M+H]^+^ | C_9_H_13_N_3_O_4_ | 2'-Deoxycytidine | Cytidine | 44721303 | 0 | 0.06 | 2.01 | 0.0074 | 0.12 | 1.62 |
| PB2 | 2.13 | 190.0503 | [M-H]^-^ | C_10_H_9_NO_3_ | 5-Hydroxyindoleacetic acid | Indoleacetic Acids | 1826 | 0.0078 | 2.47 | 1.82 | 0.0062 | 2.84 | 1.75 |
| PB3 | 4.03 | 418.2455 | [M+H]^+^ | C_16_H_31_N_7_O_6_ | Lys-Asp-Arg | Oligopeptides | 18222380 | 0.004 | 2.74 | 1.53 | 0.0072 | 2.52 | 1.6 |
| PB4 | 4.96 | 462.2716 | [M+H]^+^ | C_26_H_45_NO_7_S | Taurocholic acid | Taurine | 6675 | 0.003 | 2.55 | 1.59 | 0.0074 | 2.21 | 1.63 |
| PB5 | 5.99 | 555.2793 | [M+H]^+^ | C_25_H_46_O_11_S | SQMG (16:1/0:0) | Epoxy FA_25 | 42607389 | 0 | 3.03 | 2.04 | 0 | 2.63 | 2.19 |
| PB6 | 6.02 | 577.2866 | [M-H]^-^ | C_31_H_47_O_8_P | PA 28:7 | Phosphatidic Acids | 134764357 | 0 | 2.15 | 2.25 | 0.0001 | 2.1 | 2.16 |
| PB7 | 6.13 | 638.3048 | [M-H]^-^ | C_32_H_50_NO_10_P | PS 26:6 | Glycerophospholipids | 134728097 | 0 | 8.1 | 2.18 | 0.0001 | 7.27 | 2.15 |
| PB8 | 6.4 | 775.4089 | [M+H]^+^ | C_40_H_54_N_8_O_8_ | Angiotensin IV | Oligopeptides | 123814 | 0 | 3.86 | 2.06 | 0 | 2.98 | 2.13 |
| PB9 | 6.47 | 438.2705 | [M+H]^+^ | C_21_H_35_N_5_O_5_ | Tyr-Lys-Lys | Oligopeptides | 18232062 | 0 | 2.71 | 2.13 | 0 | 2.52 | 2.22 |
| PB10 | 6.49 | 902.4633 | [M-H]^-^ | C_44_H_61_N_11_O_10_ | Bradykinin fragment 2-9 | Bradykinin | 4140859 | 0.0001 | 5.56 | 1.97 | 0.0034 | 3.45 | 1.92 |
| PB11 | 6.55 | 482.297 | [M+H]^+^ | C_26_H_45_NO_6_S | Tauroursodeoxycholic acid | Taurochenodeoxycholic Acid | 91746279 | 0 | 2.72 | 2.11 | 0 | 2.64 | 2.21 |
| PB12 | 6.59 | 504.3105 | [M+H]^+^ | C_25_H_46_NO_7_P | LysoPE (20:3) | Plasmalogens | 53480934 | 0 | 2.74 | 2.1 | 0 | 2.69 | 2.19 |
| PB13 | 6.85 | 753.4721 | [M+H]^+^ | C_41_H_69_O_10_P | PG (13:0/22:6) | Glycerophospholipids | 52926357 | 0.0035 | 4.3 | 1.53 | 0.0076 | 3.24 | 1.57 |
| PB14 | 7.14 | 673.3959 | [M+Na]^+^ | C_36_H_58_O_10_ | ACon1_002357 | Glucosides | 23930393 | 0 | 9.3 | 2.12 | 0 | 10.76 | 2.23 |
| PB15 | 8.09 | 687.5777 | [M+H]^+^ | C_40_H_78_O_8_ | LMFA13010003 | Epoxy FA_40 | 42607337 | 0 | 2.09 | 2.12 | 0 | 2.1 | 2.21 |
| PB16 | 8.12 | 813.6749 | [M+H]^+^ | C_47_H_93_N_2_O_6_P | Sphingomyelin (d18:1/24:1) | Sphingomyelins | 44260126 | 0 | 2.02 | 1.98 | 0 | 2.05 | 2.12 |
| PB17 | 8.12 | 828.6855 | [M+H]^+^ | C_48_H_94_NO_7_P | PC (P-18:0/22:1(11Z)) | Glycerophospholipids | 52923970 | 0 | 2.62 | 2.09 | 0 | 2.59 | 2.19 |
| PB18 | 8.15 | 626.0223 | [M+H]^+^ | C_15_H_23_N_5_O_14_P_2_ | Adenosine 5'-diphosphoribose | Adenosine Diphosphate Ribose | 33576 | 0 | 2.68 | 2.13 | 0 | 2.51 | 2.21 |
| PB19 | 8.15 | 631.5271 | [M+H]^+^ | C_40_H_70_O_5_ | DG (17:2/20:2/0:0) | Glycerophospholipids | 9543749 | 0 | 3.5 | 2.13 | 0 | 3.36 | 2.22 |
| PB20 | 10.16 | 320.2552 | [M+H]^+^ | C_20_H_30_O_2_ | Eicosapentaenoic acid | Unsaturated FA | 446284 | 0.008 | 0.35 | 1.47 | 0.0007 | 0.16 | 1.87 |
| PB21 | 13.9 | 540.3288 | [M+HCOOH-H]^-^ | C_24_H_50_NO_7_P | LysoPC (16:0) | Lysophosphatidylcholines | 460602 | 0.0013 | 0.15 | 1.66 | 0.0017 | 0.18 | 1.93 |

# Table S2 The list of 81 nephrotoxicity-attenuation PBs of HK in the kidney.

| No. | RT (min) | *m/z* (Da) | Ion Type | Formula | Metabolite Name | Cluster Label | PubChem CID | CDDP to Control | | | CDDP to HK | | |
| --- | --- | --- | --- | --- | --- | --- | --- | --- | --- | --- | --- | --- | --- |
|  |  |  |  |  |  |  |  | *p* | FC | VIP | *p* | FC | VIP |
| PB1 | 1.57 | 281.1708 | [M+Na]^+^ | C_14_H_26_O_4_ | Tetradecanedioic acid | O=FA_14_1 | 13185 | 0.0001 | 0 | 1.64 | 0 | 0 | 2.15 |
| PB2 | 1.6 | 202.1187 | [M-H]^-^ | C_8_H_17_N_3_O_3_ | Glycyl-Lysine | Dipeptides | 3080576 | 0.0003 | 0.27 | 1.43 | 0.0015 | 0.34 | 1.84 |
| PB3 | 1.61 | 239.1127 | [M+Na]^+^ | C_8_H_16_N_4_O_3_ | N-a-Acetyl-L-arginine | Dipeptides | 67427 | 0 | 9.37 | 1.78 | 0.0037 | 2.95 | 1.72 |
| PB4 | 1.61 | 244.1664 | [M-H]^-^ | C_11_H_23_N_3_O_3_ | Valyl-Lysine | Dipeptides | 168058 | 0.0001 | 0.09 | 1.48 | 0.0034 | 0.15 | 1.75 |
| PB5 | 1.61 | 253.13 | [M-H]^-^ | C_11_H_18_N_4_O_3_ | Valyl-Histidine | Dipeptides | 7408624 | 0.0001 | 0.18 | 1.5 | 0.0073 | 0.36 | 1.65 |
| PB6 | 1.61 | 273.1564 | [M-H]^-^ | C_11_H_22_N_4_O_4_ | Lysyl-Glutamine | Dipeptides | 196305 | 0.0001 | 0.1 | 1.5 | 0.0098 | 0.17 | 1.66 |
| PB7 | 1.61 | 274.141 | [M-H]^-^ | C_11_H_21_N_3_O_5_ | Epsilon-(gamma-Glutamyl)-lysine | Dipeptides | 7015684 | 0 | 0 | 1.52 | 0.0059 | 0 | 1.72 |
| PB8 | 1.63 | 184.0726 | [M]^+^ | C_5_H_15_NO_4_P | Phosphorylcholine | Choline | 1014 | 0 | 3.54 | 1.79 | 0.0004 | 3.54 | 1.95 |
| PB9 | 1.63 | 327.0778 | [M+Na]^+^ | C_11_H_16_N_2_O_8_ | N-Acetylaspartylglutamic acid | Dipeptides | 5255 | 0 | 78.32 | 1.81 | 0 | ∞ | 2.14 |
| PB10 | 1.64 | 206.0543 | [M+H]^+^ | C_10_H_7_NO_4_ | Xanthurenic acid | Xanthurenates | 5699 | 0.0004 | 10.37 | 1.57 | 0.0055 | 5.81 | 1.74 |
| PB11 | 1.74 | 247.04 | [M-H]^-^ | C_8_H_13_N_2_O_5_P | Pyridoxamine 5'-phosphate | - | 1053 | 0.002 | 0.23 | 1.28 | 0.0022 | 0.46 | 1.7 |
| PB12 | 1.77 | 274.1046 | [M-H]^-^ | C_10_H_17_N_3_O_6_ | (5S,6S)-5-Methyl-2'-deoxycytidine glycol | - | 6426867 | 0.0001 | 0.14 | 1.47 | 0.0035 | 0.2 | 1.67 |
| PB13 | 1.78 | 245.1142 | [M-H]^-^ | C_10_H_18_N_2_O_5_ | Valyl-Glutamate | Dipeptides | 40520340 | 0.0008 | 0.29 | 1.36 | 0.0005 | 0.43 | 1.84 |
| PB14 | 1.79 | 134.032 | [M+H]^+^ | C_4_H_7_NO_2_S | thioproline | Sorbitol | 5081328 | 0.0026 | 0.07 | 1.41 | 0.0042 | 0.09 | 1.79 |
| PB15 | 1.81 | 231.0988 | [M-H]^-^ | C_9_H_16_N_2_O_5_ | Boc-L-isoasparagine | Oligopeptides | 7018765 | 0.0002 | 0.34 | 1.43 | 0.0035 | 0.49 | 1.65 |
| PB16 | 1.82 | 261.0729 | [M-H]^-^ | C_9_H_14_N_2_O_7_ | (5R,6S)-2'-Deoxyuridine glycol | - | 6426865 | 0.0081 | 0.47 | 1.17 | 0.0085 | 0.48 | 1.55 |
| PB17 | 1.82 | 288.1207 | [M-H]^-^ | C_11_H_19_N_3_O_6_ | Ophthalmic acid | Oligopeptides | 7018721 | 0.0004 | 0.2 | 1.42 | 0.003 | 0.27 | 1.71 |
| PB18 | 1.83 | 174.0427 | [M-H]^-^ | C_6_H_9_NO_5_ | N-Acetyl-L-aspartic acid | Dipeptides | 65065 | 0.0001 | 8.58 | 1.5 | 0.0064 | 3.35 | 1.69 |
| PB19 | 1.83 | 260.1317 | [M-H]^-^ | C_9_H_19_N_5_O_4_ | Arginyl-Serine | Dipeptides | 7021454 | 0.0019 | 0.03 | 1.31 | 0.0033 | 0.07 | 1.74 |
| PB20 | 1.83 | 302.1358 | [M-H]^-^ | C_12_H_21_N_3_O_6_ | Nicotianamine | - | 49852318 | 0 | 0 | 1.67 | 0 | 0 | 2.13 |
| PB21 | 1.87 | 298.0694 | [M-H]^-^ | C_9_H_18_NO_8_P | Phosphopantothenic Acid | - | 41635 | 0 | 0.32 | 1.57 | 0.0008 | 0.41 | 1.8 |
| PB22 | 1.96 | 203.1387 | [M+H]^+^ | C_9_H_18_N_2_O_3_ | Alanyl-Isoleucine | Dipeptides | 7408078 | 0.0015 | 0.15 | 1.47 | 0.0005 | 0.14 | 1.98 |
| PB23 | 2.07 | 175.047 | [M+Na]^+^ | C_7_H_8_N_2_O_2_ | N-(Hydroxymethyl)nicotinamide | Pyridines | 77116 | 0.0002 | 27.08 | 1.62 | 0.0039 | 5.3 | 1.8 |
| PB24 | 2.17 | 310.1743 | [M+H]^+^ | C_15_H_23_N_3_O_4_ | Tyrosyl-Lysine | Dipeptides | 7021830 | 0 | 0 | 1.87 | 0.001 | 0 | 1.82 |
| PB25 | 2.19 | 239.1009 | [M+H]^+^ | C_11_H_14_N_2_O_4_ | Tyrosyl-Glycine | Dipeptides | 7021853 | 0 | 0 | 1.83 | 0.001 | 0 | 1.87 |
| PB26 | 2.19 | 293.114 | [M-H]^-^ | C_14_H_18_N_2_O_5_ | Phenylalanyl-Glutamate | Dipeptides | 151134 | 0.0037 | 0.3 | 1.24 | 0.0056 | 0.45 | 1.66 |
| PB27 | 2.2 | 367.1496 | [M+H]^+^ | C_17_H_22_N_2_O_7_ | Tetrahydropentoxyline | Indoles | 21125948 | 0 | 4.7 | 1.8 | 0.0012 | 2.09 | 1.96 |
| PB28 | 2.22 | 192.0645 | [M+H]^+^ | C_10_H_9_NO_3_ | 5-Hydroxyindoleacetic acid | Indoles | 1826 | 0 | ∞ | 1.87 | 0 | 10.69 | 2.16 |
| PB29 | 2.23 | 209.0909 | [M+H]^+^ | C_10_H_12_N_2_O_3_ | L-Kynurenine | Amino Acids | 161166 | 0.0012 | 14.36 | 1.51 | 0.002 | ∞ | 1.88 |
| PB30 | 2.26 | 403.2193 | [M+H]^+^ | C_16_H_30_N_6_O_6_ | Ile-Asp-Arg | Oligopeptides | 18221659 | 0.0016 | 0.04 | 1.44 | 0.0034 | 0.05 | 1.83 |
| PB31 | 2.28 | 258.1457 | [M-H]^-^ | C_11_H_21_N_3_O_4_ | Isoleucyl-Glutamine | Dipeptides | 7020101 | 0.0053 | 0.35 | 1.22 | 0.0082 | 0.39 | 1.67 |
| PB32 | 2.3 | 246.1444 | [M+H]^+^ | C_10_H_19_N_3_O_4_ | Isoleucyl-Asparagine | Oligopeptides | 7016079 | 0.0054 | 0 | 1.33 | 0.0066 | 0 | 1.75 |
| PB33 | 2.33 | 123.0545 | [M+H]^+^ | C_6_H_6_N_2_O | Niacinamide | Pyridines | 936 | 0 | 0.09 | 1.79 | 0 | 0.11 | 2.1 |
| PB34 | 2.37 | 187.1095 | [M-H]^-^ | C_8_H_16_N_2_O_3_ | Glycyl-L-norleucine | Dipeptides | 273080 | 0.002 | 0.41 | 1.29 | 0.001 | 0.47 | 1.79 |
| PB35 | 2.38 | 189.1229 | [M+H]^+^ | C_8_H_16_N_2_O_3_ | Alanyl-Valine | Dipeptides | 6992383 | 0.0006 | 0.39 | 1.52 | 0.0014 | 0.46 | 1.89 |
| PB36 | 2.5 | 263.1407 | [M+H]^+^ | C_14_H_18_N_2_O_3_ | L-phenylalanyl-L-proline | Dipeptides | 44369313 | 0.0003 | 0.16 | 1.57 | 0.0099 | 0.29 | 1.68 |
| PB37 | 2.53 | 118.0863 | [M+H]^+^ | C_5_H_11_NO_2_ | 5-Aminopentanoic acid | Amino Acids, Neutral | 138 | 0 | 0 | 1.66 | 0.0007 | 0 | 1.91 |
| PB38 | 2.67 | 407.1713 | [2M-H]^-^ | C_11_H_12_N_2_O_2_ | L-Tryptophan | Indoles | 6305 | 0.002 | 0.38 | 1.29 | 0.0021 | 0.42 | 1.66 |
| PB39 | 3.05 | 262.1165 | [M+H]^+^ | C_13_H_15_N_3_O_3_ | Glycyl-Tryptophan | Dipeptides | 1551340 | 0.0087 | 0.39 | 1.26 | 0.008 | 0.48 | 1.67 |
| PB40 | 3.54 | 529.3335 | [M+H]^+^ | C_35_H_44_O_4_ | LMPK12120242 | Chalcones | 42607600 | 0 | 0.28 | 1.75 | 0.0045 | 0.4 | 1.75 |
| PB41 | 5.19 | 300.1928 | [M-H]^-^ | C_14_H_27_N_3_O_4_ | Ile-Gly-Ile | Dipeptides | 9879428 | 0.002 | 0.06 | 1.3 | 0.0095 | 0.06 | 1.63 |
| PB42 | 5.76 | 619.3266 | [M+H]^+^ | C_35_H_46_N_4_O_4_S | BILA 2185BS | Pyridines | 461204 | 0 | 0.08 | 1.82 | 0.004 | 0.18 | 1.81 |
| PB43 | 5.85 | 475.2766 | [M+H]^+^ | C_21_H_38_N_4_O_8_ | Amastatin | Peptides | 439518 | 0 | 0.12 | 1.8 | 0.0007 | 0.16 | 1.96 |
| PB44 | 5.9 | 300.1929 | [M-H]^-^ | C_14_H_27_N_3_O_4_ | Leu-Gly-Leu | Dipeptides | 259324 | 0 | 0.29 | 1.56 | 0.0025 | 0.37 | 1.73 |
| PB45 | 6.19 | 844.4583 | [M+H]^+^ | C_43_H_65_N_5_O_10_S | Tubulysin A | Oligopeptides | 12134544 | 0.0049 | 0 | 1.34 | 0.0075 | 0 | 1.71 |
| PB46 | 6.21 | 767.465 | [M-H]^-^ | C_38_H_73_O_13_P | PI 29:0 | Phosphatidylinositols | 134768939 | 0 | 0.11 | 1.64 | 0.009 | 0.16 | 1.62 |
| PB47 | 8.27 | 244.1377 | [M-H]^-^ | C_9_H_19_N_5_O_3_ | Alanyl-Arginine | Dipeptides | 446132 | 0 | 6.81 | 1.66 | 0.0019 | 2.2 | 1.8 |
| PB48 | 8.92 | 766.5363 | [M+H]^+^ | C_43_H_76_NO_8_P | PC (15:0/20:5) | Phosphatidylcholines | 52922332 | 0.0035 | 8.3 | 1.38 | 0.0034 | ∞ | 1.72 |
| PB49 | 9.95 | 280.2628 | [M+H]^+^ | C_18_H_33_NO | Linoleamide | Linoleic Acids | 6435901 | 0.0004 | 13.66 | 1.59 | 0.0029 | 7.36 | 1.83 |
| PB50 | 9.97 | 353.1975 | [M-H]^-^ | C_19_H_30_O_6_ | LMFA01040028 | - | 5282868 | 0 | 38.17 | 1.66 | 0 | 23.6 | 2.01 |
| PB51 | 10.37 | 279.2311 | [M+Na]^+^ | C_16_H_32_O_2_ | Palmitic acid | Saturated FA | 985 | 0.0004 | 0.34 | 1.57 | 0.0055 | 0.44 | 1.72 |
| PB52 | 10.73 | 555.2923 | [M+H]^+^ | C_28_H_38_N_6_O_6_ | Leucine enkephalinamide | Oligopeptides | 6427047 | 0 | 0.09 | 1.77 | 0.0045 | 0.23 | 1.78 |
| PB53 | 10.73 | 707.4661 | [M+H]^+^ | C_40_H_67_O_8_P | PA (15:0/22:6) | Phosphatidic Acids | 52928751 | 0 | 0.1 | 1.68 | 0.0094 | 0.26 | 1.69 |
| PB54 | 10.8 | 303.2306 | [M+H]^+^ | C_20_H_30_O_2_ | Eicosapentaenoic acid | UnSaturated FA | 446284 | 0.0013 | 0.09 | 1.48 | 0.0015 | 0.09 | 1.84 |
| PB55 | 10.84 | 600.4646 | [M+H]^+^ | C_34_H_65_NO_5_S | LMSP00000003 | - | 42608341 | 0.0003 | ∞ | 1.6 | 0.0017 | 72.57 | 1.88 |
| PB56 | 10.86 | 571.2873 | [M-H]^-^ | C_25_H_49_O_12_P | PI (16:0/0:0) | Phosphatidylinositols | 71296207 | 0 | 0.2 | 1.55 | 0.0048 | 0.47 | 1.65 |
| PB57 | 11.56 | 502.2912 | [M+H]^+^ | C_25_H_44_NO_7_P | LysoPE (20:4/0:0) | Phosphatidylethanolamines | 42607465 | 0 | 0 | 1.85 | 0 | 0 | 2.26 |
| PB58 | 12.94 | 401.2647 | [M+Na]^+^ | C_23_H_38_O_4_ | MG (20:4/0:0/0:0) | Glycerides | 16019980 | 0.0004 | 7.33 | 1.55 | 0.008 | 3.56 | 1.61 |
| PB59 | 13.23 | 572.3697 | [M+H]^+^ | C_30_H_54_NO_7_P | LysoPC (22:4) | Unsaturated_Lysophosphatidylcholines | 52924039 | 0.0001 | 15.74 | 1.68 | 0.0058 | 3.57 | 1.76 |
| PB60 | 13.53 | 377.265 | [M+H]^+^ | C_23_H_36_O_4_ | MG (20:5/0:0/0:0) | Glycerides | 53480988 | 0.0001 | 12.4 | 1.62 | 0.004 | 4.73 | 1.76 |
| PB61 | 13.78 | 518.3207 | [M+H]^+^ | C_26_H_48_NO_7_P | LysoPC (18:3) | Unsaturated_Lysophosphatidylcholines | 24779469 | 0.0002 | 0 | 1.59 | 0 | 0 | 2.18 |
| PB62 | 13.92 | 548.3674 | [M+H]^+^ | C_28_H_55_NO_7_P | LysoPC (20:2) | Unsaturated_Lysophosphatidylcholines | 52924053 | 0.0001 | 7.43 | 1.67 | 0.0029 | 3.02 | 1.81 |
| PB63 | 14.06 | 328.2826 | [M+H]^+^ | C_19_H_37_NO_3_ | N-palmitoyl alanine | Dipeptides | 14961184 | 0 | 0.06 | 1.69 | 0.0008 | 0.1 | 1.95 |
| PB64 | 14.63 | 496.3371 | [M+H]^+^ | C_24_H_50_NO_7_P | LysoPC (16:0) | Saturated_Lysophosphatidylcholines | 460602 | 0.0026 | 0 | 1.43 | 0.0007 | 0 | 1.92 |
| PB65 | 14.88 | 481.3467 | [M+H]^+^ | C_29_H_46_F_2_O_3_ | Dmdfdh-D3 | Hydroxycholecalciferols | 6439159 | 0.0004 | 0 | 1.56 | 0.0019 | 0 | 1.85 |
| PB66 | 14.9 | 480.3429 | [M+H]^+^ | C_24_H_50_NO_6_P | LysoPC (P-16:0) | Lysophospholipids | 10917802 | 0 | 0 | 1.75 | 0.0081 | 0 | 1.64 |
| PB67 | 15.1 | 510.3524 | [M+H]^+^ | C_25_H_52_NO_7_P | LysoPC (17:0) | Saturated_Lysophosphatidylcholines | 24779463 | 0 | 0 | 1.69 | 0.0005 | 0 | 1.95 |
| PB68 | 15.79 | 350.3043 | [M+H]^+^ | C_22_H_39_NO_2_ | Dihomo-gamma-Linolenoyl ethanolamide | Polyunsaturated Alkamides | 5282272 | 0.0001 | 4.32 | 1.63 | 0.0099 | 2.26 | 1.61 |
| PB69 | 15.88 | 482.3589 | [M+H]^+^ | C_24_H_52_NO_6_P | PC (O-16:0/0:0) | Saturated_Lysophosphatidylcholines | 162126 | 0.0042 | 0.01 | 1.35 | 0.0009 | 0 | 1.91 |
| PB70 | 16.53 | 104.1078 | [M]^+^ | C_5_H_14_NO^+^ | Choline | Ethanolamines | 305 | 0.0014 | 0.2 | 1.46 | 0.0025 | 0.24 | 1.86 |
| PB71 | 16.7 | 546.3507 | [M+H]^+^ | C_28_H_52_NO_7_P | LysoPC (20:3) | Unsaturated_Lysophosphatidylcholines | 53480467 | 0 | 0 | 1.78 | 0.0023 | 0 | 1.85 |
| PB72 | 16.76 | 465.3033 | [M-H]^-^ | C_27_H_46_O_4_S | Cholesterol sulfate | Cholesterol Esters | 65076 | 0 | 0.21 | 1.6 | 0.0095 | 0.33 | 1.53 |
| PB73 | 18.2 | 724.526 | [M+H]^+^ | C_41_H_74_NO_7_P | PE (18:3/P-18:1) | Phosphatidylethanolamines | 53479683 | 0.0097 | 0.13 | 1.25 | 0.002 | 0 | 1.79 |
| PB74 | 18.23 | 753.5806 | [M+Na]^+^ | C_41_H_83_N_2_O_6_P | N-Octadecanoyl-D-sphingosine-1-phosphocholine | Sphingomyelins | 5353953 | 0.0055 | 4.81 | 1.31 | 0.0002 | 29.28 | 1.94 |
| PB75 | 18.54 | 724.5252 | [M+H]^+^ | C_41_H_74_NO_7_P | PE (P-18:0/18:4) | Phosphatidylethanolamines | 52925082 | 0.001 | 0 | 1.49 | 0.002 | 0 | 1.79 |
| PB76 | 18.55 | 736.4911 | [M-H]^-^ | C_41_H_72_NO_8_P | PE (16:0/20:5) | Phosphatidylethanolamines | 52924919 | 0.0045 | 2.19 | 1.26 | 0.0058 | 2.21 | 1.56 |
| PB77 | 18.88 | 764.5198 | [M+H]^+^ | C_43_H_74_NO_8_P | PE (18:1/20:5) | Phosphatidylethanolamines | 53479637 | 0.002 | 2.16 | 1.47 | 0.0059 | 2.16 | 1.73 |
| PB78 | 18.94 | 826.5357 | [M+Na]^+^ | C_46_H_78_NO_8_P | PC (16:1/22:6) | Phosphatidylcholines | 24778773 | 0.0001 | 3.79 | 1.65 | 0.0015 | 3.87 | 1.85 |
| PB79 | 19.54 | 740.5203 | [M+H]^+^ | C_41_H_74_NO_8_P | PE (18:2/18:2) | Phosphatidylethanolamines | 9546812 | 0.0008 | 2.57 | 1.52 | 0.0085 | 2.04 | 1.61 |
| PB80 | 20.16 | 874.5593 | [M+HCOOH-H]^-^ | C_48_H_80_NO_8_P | PC (20:4/20:4) | Phosphatidylcholines | 24779076 | 0.0017 | 2.13 | 1.32 | 0.0016 | 2.69 | 1.68 |
| PB81 | 20.71 | 748.5815 | [M+H]^+^ | C_41_H_82_NO_8_P | PC (15:0/18:0) | Phosphatidylcholines | 11787533 | 0.0004 | 0 | 1.56 | 0.009 | 0 | 1.63 |

# Table S3 The list of 68 nephrotoxicity-attenuation PBs of HK in the urine based on LC-MS.

| No. | RT (min) | *m/z* (Da) | Ion Type | Formula | Metabolite Name | Cluster Label | PubChem CID | CDDP to Control | | | CDDP to HK | | |
| --- | --- | --- | --- | --- | --- | --- | --- | --- | --- | --- | --- | --- | --- |
|  |  |  |  |  |  |  |  | *p* | FC | VIP | *p* | FC | VIP |
| rPB1 | 1.76 | 176.0665 | [M+H]^+^ | C_10_H_9_NO_2_ | 3-Indoleacetic acid | Indoleacetic Acids | 802 | 0 | ∞ | 1.03 | 0 | 15.19 | 1.12 |
| rPB2 | 1.76 | 209.0297 | [M-H]^-^ | C_6_H_8_O_7_ | D-Saccharic acid 1,4-lactone | Sugar Acids | 122306 | 0 | 4.28 | 1.51 | 0 | 2.72 | 1.75 |
| rPB3 | 1.77 | 146.0446 | [M+H]^+^ | C_5_H_7_NO_4_ | 2-Keto-glutaramic acid | Ketoglutaric Acids | 48 | 0 | 57.3 | 1.24 | 0 | 4.58 | 1.2 |
| rPB4 | 1.81 | 187.0722 | [M-H]^-^ | C_7_H_12_N_2_O_4_ | L-glycyl-L-hydroxyproline | Dipeptides | 259579 | 0 | 0.14 | 1.5 | 0 | 0.17 | 1.6 |
| rPB5 | 1.81 | 273.1076 | [M+H]^+^ | C_11_H_16_N_2_O_6_ | 5-Ethyluridine (A) | Pyrimidine Nucleosides | 146980 | 0 | 8.94 | 3.29 | 0 | 2.2 | 2.79 |
| rPB6 | 1.82 | 259.0919 | [M+H]^+^ | C_10_H_14_N_2_O_6_ | 3-Methyluridine | Pyrimidine Nucleosides | 99592 | 0 | 5.69 | 6.2 | 0 | 28.91 | 4.26 |
| rPB7 | 1.83 | 167.0213 | [M-H]^-^ | C_5_H_4_N_4_O_3_ | Uric acid (A) | Purinones | 1175 | 0 | 0.19 | 1.51 | 0 | 0.27 | 1.77 |
| rPB8 | 1.86 | 369.0654 | [M+FA-H]^-^ | C_12_H_18_O_13_ | Digalacturonic acid | Disaccharides | 439694 | 0.0002 | 13.51 | 1.2 | 0 | 6.43 | 1.3 |
| rPB9 | 1.88 | 231.0978 | [M-H]^-^ | C_9_H_16_N_2_O_5_ | Val-Asp (A) | Dipeptides | 7009608 | 0 | 0.02 | 1.51 | 0 | 0.04 | 1.75 |
| rPB10 | 1.92 | 175.0232 | [M+H]^+^ | C_6_H_6_O_6_ | cis-Aconitic acid | Tricarboxylic Acids | 643757 | 0 | 61.73 | 3.18 | 0 | 3.19 | 2.9 |
| rPB11 | 1.92 | 193.0341 | [M+H]^+^ | C_6_H_8_O_7_ | Citric acid | Tricarboxylic Acids | 19782904 | 0 | 821.48 | 3.88 | 0 | 3.34 | 3.57 |
| rPB12 | 2.04 | 133.0127 | [M-H]^-^ | C_4_H_6_O_5_ | DL-Malic acid | Malates | 20130941 | 0 | 2.03 | 1.44 | 0 | 2.24 | 1.7 |
| rPB13 | 2.35 | 150.0768 | [M+H]^+^ | C_6_H_7_N_5_ | 3-Methyladenine | - | 1673 | 0 | 66.65 | 1.92 | 0 | 10.78 | 2.1 |
| rPB14 | 2.38 | 147.0286 | [M+H]^+^ | C_5_H_6_O_5_ | Oxoglutaric acid | Ketoglutaric Acids | 51 | 0 | ∞ | 1.11 | 0 | 4.91 | 1.11 |
| rPB15 | 2.39 | 141.0658 | [M+H]^+^ | C_6_H_8_N_2_O_2_ | Methylimidazoleacetic acid | Imidazoles | 75810 | 0 | 2.74 | 2.13 | 0 | 2.51 | 2.29 |
| rPB16 | 2.39 | 167.0208 | [M-H]^-^ | C_5_H_4_N_4_O_3_ | Uric acid (B) | Purinones | 1175 | 0 | 0.12 | 1.45 | 0 | 0.19 | 1.7 |
| rPB17 | 2.39 | 231.0985 | [M-H]^-^ | C_9_H_16_N_2_O_5_ | Val-Asp (B) | Dipeptides | 7009608 | 0 | 0.05 | 1.47 | 0 | 0.07 | 1.67 |
| rPB18 | 2.39 | 273.1078 | [M+H]^+^ | C_11_H_16_N_2_O_6_ | 5-Ethyluridine (B) | Pyrimidine Nucleosides | 146980 | 0 | 12.04 | 5.48 | 0 | 3.36 | 5.34 |
| rPB19 | 2.45 | 181.0362 | [M-H]^-^ | C_6_H_6_N_4_O_3_ | 1-Methyluric acid | Purinones | 69726 | 0 | 0.03 | 1.5 | 0 | 0.02 | 1.85 |
| rPB20 | 2.47 | 245.0761 | [M+H]^+^ | C_9_H_12_N_2_O_6_ | uridine | Pyrimidine Nucleosides | 6029 | 0 | ∞ | 2.97 | 0 | 261.1 | 3.39 |
| rPB21 | 2.67 | 164.0576 | [M-H]^-^ | C_6_H_7_N_5_O | 7-Methylguanine | Purinones | 11361 | 0 | 0 | 1.53 | 0 | 0 | 1.83 |
| rPB22 | 2.72 | 255.1331 | [M+H]^+^ | C_12_H_18_N_2_O_4_ | L-Furosine | - | 14497053 | 0.0001 | ∞ | 1.55 | 0 | ∞ | 1.78 |
| rPB23 | 2.74 | 159.0759 | [M+H]^+^ | C_6_H_10_N_2_O_3_ | 5-Hydroxyectoine | Amino Acids | 12011795 | 0 | ∞ | 1.15 | 0 | ∞ | 1.32 |
| rPB24 | 2.88 | 263.0226 | [M-H]^-^ | C_9_H_12_O_7_S | 3-Methoxy-4-hydroxyphenylglycol sulfate | Benzyl Alcohols | 3035420 | 0 | 0 | 1.54 | 0 | 0 | 1.55 |
| rPB25 | 2.89 | 418.1087 | [M-H]^-^ | C_14_H_25_NO_11_ | Galacto-N-biose | Antigens, Neoplasm | 441248 | 0.0077 | 0.1 | 1.16 | 0 | 0.04 | 1.53 |
| rPB26 | 2.9 | 188.0546 | [M-H]^-^ | C_7_H_11_NO_5_ | N-Acetyl-L-glutamic acid | Glutamates | 70914 | 0.0008 | ∞ | 1.1 | 0 | 46.8 | 1.43 |
| rPB27 | 2.9 | 216.0984 | [M-H]^-^ | C_8_H_15_N_3_O_4_ | Glutaminylalanine | Oligopeptides | 9813211 | 0.0008 | ∞ | 1.1 | 0 | ∞ | 1.43 |
| rPB28 | 2.93 | 173.0091 | [M-H]^-^ | C_6_H_6_O_6_ | trans-Aconitic acid | Tricarboxylic Acids | 444212 | 0 | 113.29 | 1.33 | 0 | 90.38 | 1.69 |
| rPB29 | 3.01 | 136.0757 | [M+H]^+^ | C_8_H_9_NO | 2-Phenylacetamide | Benzeneacetamides | 7680 | 0 | ∞ | 1.07 | 0 | ∞ | 1.23 |
| rPB30 | 3.02 | 126.0663 | [M+H]^+^ | C_5_H_7_N_3_O | 5-Methylcytosine | Cytosine | 65040 | 0 | ∞ | 2.44 | 0 | 1188.72 | 2.79 |
| rPB31* | 3.03 | 182.0805 | [M+H]^+^ | C_9_H_11_NO_3_ | L-Tyrosine | Amino Acids, Cyclic | 6057 | 0 | ∞ | 2.34 | 0 | ∞ | 2.68 |
| rPB32 | 3.14 | 160.1078 | [M+H]^+^ | C_6_H_13_N_3_O_2_ | delta-Guanidinovaleric acid | Valerates | 160464 | 0 | ∞ | 1.59 | 0 | ∞ | 1.81 |
| rPB33 | 3.19 | 116.0704 | [M+H]^+^ | C_5_H_9_NO_2_ | Proline | Amino Acids, Cyclic | 145742 | 0 | 98.75 | 1.49 | 0 | 25.94 | 1.68 |
| rPB34 | 3.22 | 265.0234 | [M-H]^-^ | C_5_H_14_O_7_P_2_ | Isopentyl pyrophosphate | - | 448400 | 0 | ∞ | 1.46 | 0 | 9.01 | 1.64 |
| rPB35* | 3.23 | 132.1017 | [M+H]^+^ | C_6_H_13_NO_2_ | Isoleucine | Amino Acids | 6306 | 0 | ∞ | 2.14 | 0 | ∞ | 2.45 |
| rPB36 | 3.38 | 157.0137 | [M-H]^-^ | C_9_H_12_O_6_ | 5,6-Isopropylidene-L-ascorbic acid | - | 54691418 | 0 | ∞ | 1.53 | 0 | 33.2 | 1.95 |
| rPB37 | 3.38 | 307.0230 | [M-H]^-^ | C_9_H_13_N_2_O_8_P | dUMP | Pyrimidine Nucleosides | 65063 | 0.0011 | ∞ | 1.1 | 0 | ∞ | 1.39 |
| rPB38 | 3.48 | 101.0243 | [M-H]^-^ | C_4_H_6_O_3_ | 2-Oxobutyrate | Butyrates | 58 | 0 | ∞ | 1.35 | 0 | ∞ | 1.73 |
| rPB39 | 3.52 | 125.0347 | [M-H]^-^ | C_5_H_6_N_2_O_2_ | 4-Imidazoleacetic acid | Imidazoles | 28305488 | 0.0005 | 0 | 1.25 | 0 | 0 | 1.62 |
| rPB40 | 3.57 | 215.1042 | [M-H]^-^ | C_9_H_16_N_2_O_4_ | Thr-Pro | Oligopeptides | 11937736 | 0 | 0 | 1.51 | 0 | 0 | 1.62 |
| rPB41 | 3.65 | 144.0654 | [M-H]^-^ | C_6_H_11_NO_3_ | 4-Acetamidobutanoic acid | Glutamates | 18189 | 0 | 0 | 1.51 | 0 | 0 | 1.9 |
| rPB42 | 3.69 | 246.0206 | [M-H]^-^ | C_8_H_10_NO_6_P | Pyridoxal phosphate | Pyridines | 1051 | 0 | ∞ | 1.3 | 0 | 14.75 | 1.58 |
| rPB43 | 4.01 | 187.1071 | [M-H]^-^ | C_8_H_16_N_2_O_3_ | N-Alpha-acetyllysine | Dipeptides | 192590 | 0 | 0.01 | 1.44 | 0.01 | 0.01 | 1.17 |
| rPB44 | 4.01 | 230.1140 | [M-H]^-^ | C_9_H_17_N_3_O_4_ | Asparaginyl-Valine | Dipeptides | 7019992 | 0 | 0 | 1.53 | 0 | 0 | 1.61 |
| rPB45 | 4.04 | 154.0509 | [M+H]^+^ | C_7_H_7_NO_3_ | 3-Hydroxyanthranilic acid | ortho-Aminobenzoates | 86 | 0 | 0 | 1.5 | 0 | 0 | 2.14 |
| rPB46* | 4.38 | 182.0462 | [M-H]^-^ | C_8_H_9_NO_4_ | 4-Pyridoxic acid | Pyridines | 6723 | 0.006 | 0.21 | 1.1 | 0 | 0.18 | 1.54 |
| rPB47 | 4.45 | 288.1201 | [M-H]^-^ | C_11_H_19_N_3_O_6_ | Ophthalmic acid | Oligopeptides | 76138099 | 0.0014 | ∞ | 1.07 | 0 | ∞ | 1.38 |
| rPB48 | 4.56 | 257.1128 | [M+H]^+^ | C_11_H_16_N_2_O_5_ | 5'-O-Methylthymidine | Pyrimidine Nucleosides | 14483474 | 0.0002 | 8.25 | 2.14 | 0 | 53.92 | 2.69 |
| rPB49 | 4.57 | 153.0657 | [M+H]^+^ | C_7_H_8_N_2_O_2_ | N1-Methyl-2-pyridone-5-carboxamide | Pyridines | 69698 | 0 | 9.94 | 7.51 | 0 | 10.94 | 8.62 |
| rPB50 | 4.58 | 151.0615 | [M+H]^+^ | C_6_H_6_N_4_O | 1-Methylhypoxanthine | Purinones | 70765 | 0 | 30.89 | 6.14 | 0 | 28.9 | 6.99 |
| rPB51 | 4.76 | 142.0512 | [M-H]^-^ | C_6_H_11_NO_4_ | DL-2-Methylglutamic acid | Glutamates | 95440 | 0.0005 | 0 | 1.22 | 0.01 | 0 | 1.38 |
| rPB52 | 5.32 | 212.0535 | [M-H]^-^ | C_6_H_15_NO_5_S | N,N-Bis(2-hydroxyethyl)-2-aminoethanesulfonic acid | - | 6993707 | 0 | 0.15 | 1.33 | 0 | 0.07 | 1.52 |
| rPB53 | 5.34 | 177.0753 | [M-H]^-^ | C_7_H_14_O_5_ | 2-[2-(2-Methoxyethoxy)ethoxy]acetic acid | Acetates | 85241 | 0 | ∞ | 1.52 | 0 | ∞ | 1.94 |
| rPB54 | 5.75 | 258.0053 | [M-H]^-^ | C_6_H_14_NO_8_P | Galactosamine 1-phosphate | Galactosephosphates | 3036672 | 0 | 12.28 | 1.42 | 0 | 21.48 | 1.82 |
| rPB55 | 5.81 | 166.0862 | [M+H]^+^ | C_9_H_11_NO_2_ | L-Phenylalanine | Amino Acids, Cyclic | 6140 | 0 | 539.35 | 2.55 | 0 | 312.89 | 2.91 |
| rPB56 | 6.68 | 214.1065 | [M-H]^-^ | C_10_H_17_NO_4_ | N-alpha-(tert-Butoxycarbonyl)-L-proline | - | 688022 | 0 | ∞ | 1.45 | 0 | 3.9 | 1.7 |
| rPB57 | 6.74 | 210.0733 | [M+H]^+^ | C_10_H_11_NO_4_ | N-Benzyloxycarbonylglycine | Dipeptides | 14349 | 0.0004 | 58.22 | 1.03 | 0 | 43.48 | 1.16 |
| rPB58 | 6.76 | 188.0913 | [M+H]^+^ | C_8_H_13_NO_4_ | 2-Keto-6-acetamidocaproate | Aminocaproates | 194080 | 0.0006 | 16.29 | 1.33 | 0 | 28.46 | 1.55 |
| rPB59 | 6.97 | 158.0815 | [M+H]^+^ | C_7_H_11_NO_3_ | 3-Methylcrotonylglycine | - | 169485 | 0 | 359.87 | 3.31 | 0 | 411.36 | 3.77 |
| rPB60 | 8.99 | 264.0538 | [M+H]^+^ | C_9_H_13_NSO_6_ | Normetanephrine sulfate | - | - | 0.0001 | 3.32 | 3.3 | 0 | 3.29 | 3.75 |
| rPB61 | 9.14 | 261.0077 | [M-H]^-^ | C_9_H_10_O_7_S | Homovanillic acid sulfate | - | 29981063 | 0.0008 | 80.56 | 1.13 | 0 | ∞ | 1.43 |
| rPB62 | 9.68 | 285.1456 | [M-H]^-^ | C_18_H_22_O_3_ | 16a-Hydroxyestrone | Hydroxyestrones | 115116 | 0 | ∞ | 1.33 | 0 | 10.51 | 1.7 |
| rPB63 | 9.86 | 190.0503 | [M-H]^-^ | C_10_H_9_NO_3_ | 5-Hydroxyindole-3-acetic acid | Indoleacetic Acids | 1826 | 0 | 3.9 | 1.46 | 0 | 2.67 | 1.85 |
| rPB64 | 9.95 | 249.0874 | [M-H]^-^ | C_9_H_18_N_2_O_4_S | Methionyl-Threonine | Dipeptides | 7021821 | 0 | ∞ | 1.52 | 0 | 7.32 | 1.78 |
| rPB65 | 10.43 | 230.0785 | [M+H]^+^ | C_6_H_15_NO_6_S | N-TrI (hydroxymethyl)methyltaurine | Sulfonic Acids | 6992013 | 0 | 9.36 | 1.22 | 0 | 2.96 | 1.1 |
| rPB66 | 10.55 | 160.0399 | [M-H]^-^ | C_9_H_7_NO_2_ | 1H-Indole-3-carboxylic acid | Indoles | 69867 | 0.0001 | 2.22 | 1.41 | 0.01 | 2.63 | 1.62 |
| rPB67 | 10.95 | 204.0666 | [M-H]^-^ | C_11_H_11_NO_3_ | Indolelactic acid | Indoles | 92904 | 0 | ∞ | 1.4 | 0 | 3.56 | 1.45 |
| rPB68 | 10.96 | 206.0816 | [M+H]^+^ | C_11_H_11_NO_3_ | 5-Methoxyindoleacetate | Indoleacetic Acids | 18986 | 0 | ∞ | 1.22 | 0 | 2.24 | 1.34 |

# Table S4 The list of 26 nephrotoxicity-attenuation PBs of HK in the urine based on HILIC-MS.

| No. | RT (min) | *m/z* (Da) | Ion Type | Formula | Metabolite Name | Cluster Label | PubChem CID | CDDP to Control | | | CDDP to HK | | |
| --- | --- | --- | --- | --- | --- | --- | --- | --- | --- | --- | --- | --- | --- |
|  |  |  |  |  |  |  |  | *p* | FC | VIP | *p* | FC | VIP |
| hPB1 | 1.87 | 201.1135 | [M-H]^-^ | C_10_H_18_O_4_ | Sebacic acid | O=FA_10_1 | 5192 | 0.0003 | 0.27 | 1.16 | 0.0099 | 0.28 | 1.23 |
| hPB2 | 1.91 | 171.0658 | [M-H]^-^ | C_8_H_12_O_4_ | trans-1,4-Cyclohexanedi-carboxyli acid | Cyclohexanes | 14106 | 0.0003 | 0.1 | 1.37 | 0.0035 | 0.09 | 1.18 |
| hPB3 | 2.43 | 205.0352 | [M-H]^-^ | C_8_H_14_O_2_S_2_ | (R)-lipoic acid | Saturated FA | 6112 | 0.001 | 0.18 | 1.1 | 0.0017 | 0.13 | 1.46 |
| hPB4* | 2.44 | 167.0214 | [M-H]^-^ | C_5_H_4_N_4_O_3_ | Uric acid (A) | Purinones | 1175 | 0 | 0.18 | 1.51 | 0.0023 | 0.27 | 1.3 |
| hPB5 | 2.53 | 191.0193 | [M-H]^-^ | C_6_H_8_O_7_ | Citric acid | Tricarboxylic Acids | 19782904 | 0 | 115.62 | 1.51 | 0.0005 | 2.63 | 1.44 |
| hPB6 | 2.54 | 111.0092 | [M-H]^-^ | C_5_H_4_O_3_ | 3-Furoic acid | - | 10268 | 0 | 1387.28 | 1.51 | 0.0014 | 2.41 | 1.35 |
| hPB7* | 2.56 | 220.118 | [M+H]^+^ | C_9_H_17_NO_5_ | Pantothenic acid | beta-Alanine | 988 | 0 | 0.39 | 1.7 | 0.002 | 0.35 | 1.24 |
| hPB8 | 3.09 | 130.0609 | [M+H]^+^ | C_9_H_7_N | isoquinoline | Isoquinolines | 8405 | 0.0079 | 3.24 | 1.22 | 0.002 | 5.89 | 1.6 |
| hPB9 | 3.28 | 124.0081 | [M-H]^-^ | C_2_H_7_NO_3_S | Taurine | Alkanesulfonic Acids | 1123 | 0 | 0.28 | 1.47 | 0.0043 | 0.45 | 1.39 |
| hPB10 | 4.95 | 308.0987 | [M-H]^-^ | C_11_H_19_NO_9_ | N-Acetylneuraminic Acid | Sialic Acids | 906 | 0 | 13.54 | 1.47 | 0 | 36.6 | 1.79 |
| hPB11 | 5.17 | 281.1122 | [M+H]^+^ | C_13_H_16_N_2_O_5_ | L-Aspartyl-L-phenylalanine | Dipeptides | 93078 | 0 | 3.48 | 1.67 | 0.0005 | 2.8 | 1.22 |
| hPB12 | 5.19 | 154.0607 | [M+Na]^+^ | C_4_H_9_N_3_O_2_ | Creatine | Guanidines | 586 | 0.0001 | 3.04 | 1.43 | 0.0038 | 2.14 | 1.01 |
| hPB13 | 7.17 | 160.0961 | [M+H]^+^ | C_7_H_13_NO_3_ | Isovalerylglycine | Dipeptides | 546304 | 0 | ∞ | 1.75 | 0 | 4.3 | 1.36 |
| hPB14 | 8.23 | 218.1376 | [M+H]^+^ | C_10_H_19_NO_4_ | Propionyl-L-carnitine | Carnitine | 107738 | 0 | 9.35 | 1.72 | 0.0005 | 3.4 | 1.24 |
| hPB15 | 10.29 | 104.0345 | [M-H]^-^ | C_3_H_7_NO_3_ | L-Serine | Amino Acids | 617 | 0.0009 | ∞ | 1.11 | 0.0011 | 50.31 | 1.34 |
| hPB16 | 10.81 | 130.0515 | [M-H]^-^ | C_5_H_9_NO_3_ | trans-4-Hydroxy-L-proline | Amino Acids, Cyclic | 5810 | 0.0013 | 3.13 | 1.05 | 0.001 | 4.02 | 1.35 |
| hPB17 | 10.91 | 344.1064 | [M-H]^-^ | C_10_H_12_N_5_O_7_P | Cyclic GMP | Guanine Nucleotides | 24316 | 0 | ∞ | 1.49 | 0 | 13.39 | 1.71 |
| hPB18 | 11.69 | 118.0614 | [M+H]^+^ | C_3_H_7_N_3_O_2_ | Guanidineacetic acid | - | 3946848 | 0.0005 | 0.19 | 1.5 | 0.0051 | 0.18 | 1.62 |
| hPB19* | 12.2 | 145.0619 | [M-H]^-^ | C_5_H_10_N_2_O_3_ | L-Glutamine | Amino Acids | 145815 | 0 | 221.12 | 1.29 | 0.0047 | 3.98 | 1.16 |
| hPB20 | 13.76 | 273.0021 | [M-H]^-^ | C_6_H_11_O_10_P | D-Glucuronic acid 1-phosphate | Gluconates | 440203 | 0 | ∞ | 1.47 | 0 | 29.46 | 1.75 |
| hPB21 | 14.81 | 187.0723 | [M-H]^-^ | C_7_H_12_N_2_O_4_ | L-glycyl-L-hydroxyproline | Dipeptides | 259579 | 0 | 0 | 1.51 | 0.0025 | 0 | 1.42 |
| hPB22 | 15.96 | 221.0602 | [M-H]^-^ | C_7_H_14_N_2_O_4_S | L-Cystathionine | Amino Acids, Sulfur | 12358847 | 0.0006 | ∞ | 1.14 | 0.0006 | ∞ | 1.36 |
| hPB23 | 16.27 | 154.0623 | [M-H]^-^ | C_6_H_9_N_3_O_2_ | L-Histidine | Amino Acids, Cyclic | 6274 | 0 | 10.64 | 1.34 | 0 | 24.02 | 1.58 |
| hPB24 | 16.5 | 204.123 | [M+H]^+^ | C_9_H_17_NO_4_ | Acetylcarnitine | Carnitine | 7045767 | 0 | 10.84 | 1.54 | 0.0012 | 3.02 | 1.5 |
| hPB25 | 19.01 | 231.1696 | [M+H]^+^ | C_11_H_22_N_2_O_3_ | DL-Leu-DL-Val | Dipeptides | 352038 | 0 | 0.2 | 1.53 | 0.004 | 0.27 | 1.14 |
| hPB26 | 19.24 | 255.1339 | [M+H]^+^ | C_12_H_18_N_2_O_4_ | L-Furosine | - | 14497053 | 0 | ∞ | 1.63 | 0.0002 | 5.93 | 1.57 |

# Table S5 HK components related to CKD according to network pharmacology.

| No. | Formula | Identification or assignment | SMILES | Degree |
| --- | --- | --- | --- | --- |
| C1 | C_21_H_20_O_13_ | Gossypetin 3-glucoside | OC[C@H]1O[C@@H](OC2=C(OC3=C(C(O)=CC(O)=C3O)C2=O)C2=CC=C(O)C(O)=C2)C(O)C(O)[C@@H]1O | 3 |
| C2 | C_21_H_18_O_13_ | Quercetin ether 3-glucoside | OCC1OC(OC2=C(OC3=C4OC(=C4O)C(=O)C3=C2O)C2=CC=C(O)C(O)=C2)C(O)C(O)C1O | 3 |
| C3 | C_21_H_20_O_13_ | Gossypetin 3'-glucoside | OC[C@H]1O[C@@H](OC2=CC(=CC=C2O)C2=C(O)C(=O)C3=C(O2)C(O)=C(O)C=C3O)C(O)C(O)[C@@H]1O | 4 |
| C5 | C_27_H_30_O_17_ | Quercetin 3,5-O-Diglucoside | OC[C@H]1OC(OC2=CC(O)=CC3=C2C(=O)C(OC2O[C@H](CO)[C@@H](O)[C@H](O)[C@H]2O)=C(O3)C2=CC=C(O)C(O)=C2)C(O)[C@@H](O)C1O | 3 |
| C6 | C_27_H_30_O_17_ | Quercetin 3,7-diglucoside | OC[C@H]1O[C@@H](OC2=CC3=C(C(O)=C2)C(=O)C(O[C@@H]2O[C@H](CO)[C@@H](O)[C@H](O)[C@H]2O)=C(O3)C2=CC=C(O)C(O)=C2)[C@H](O)[C@@H](O)[C@@H]1O | 3 |
| C7 | C_21_H_20_O_13_ | Myricetin 3-glucoside | OC[C@H]1O[C@@H](OC2=C(OC3=C(C(O)=CC(O)=C3)C2=O)C2=CC(O)=C(O)C(O)=C2)C(O)C(O)[C@@H]1O | 3 |
| C8 | C_27_H_30_O_18_ | Gossypetin-3,8-O-diglucoside | OC[C@H]1O[C@@H](OC2=C(O)C=C(O)C3=C2OC(C2=CC=C(O)C(O)=C2)=C(O[C@@H]2O[C@H](CO)[C@@H](O)[C@H](O)[C@H]2O)C3=O)[C@H](O)[C@@H](O)[C@@H]1O | 4 |
| C10 | C_27_H_30_O_17_ | Quercetin 3,3'-diglucoside | OC[C@H]1OC(OC2=CC(=CC=C2O)C2=C(O[C@@H]3O[C@H](CO)[C@@H](O)[C@H](O)[C@H]3O)C(=O)C3=C(O2)C=C(O)C=C3O)[C@H](O)[C@@H](O)[C@@H]1O | 3 |
| C11 | C_27_H_28_O_19_ | Gossypetin 8-glucuronide 3-glucoside | OC[C@H]1O[C@@H](OC2=C(OC3=C(C(O)=CC(O)=C3O[C@@H]3OC([C@@H](O)[C@H](O)C3O)C(O)=O)C2=O)C2=CC=C(O)C(O)=C2)C(O)C(O)[C@@H]1O | 3 |
| C12 | C_27_H_30_O_17_ | Quercetin 7,3'-diglucoside | OC[C@H]1O[C@@H](OC2=CC3=C(C(O)=C2)C(=O)C(O)=C(O3)C2=CC=C(O)C(OC3O[C@H](CO)[C@@H](O)[C@H](O)[C@H]3O)=C2)[C@H](O)[C@@H](O)[C@@H]1O | 4 |
| C13 | C_21_H_20_O_12_ | Quercetin 3'-O-glucoside | OC[C@H]1OC(OC2=CC(=CC=C2O)C2=C(O)C(=O)C3=C(O2)C=C(O)C=C3O)[C@H](O)[C@@H](O)[C@@H]1O | 4 |
| C14 | C_27_H_30_O_16_ | Rutin | C[C@@H]1O[C@@H](OC[C@H]2O[C@@H](OC3=C(OC4=C(C(O)=CC(O)=C4)C3=O)C3=CC=C(O)C(O)=C3)[C@H](O)[C@@H](O)[C@@H]2O)[C@H](O)[C@H](O)[C@H]1O | 3 |
| C15 | C_21_H_22_O_9_ | Liquiritin | OC[C@H]1O[C@@H](OC2=CC=C(C=C2)[C@@H]2CC(=O)C3=C(O2)C=C(O)C=C3)[C@H](O)[C@@H](O)[C@@H]1O | 5 |
| C16 | C_27_H_30_O_18_ | Gossypetin 3-sophoroside | OCC1O[C@@H](OC2C(O)[C@H](O)C(CO)O[C@H]2OC2=C(OC3=C(C(O)=CC(O)=C3O)C2=O)C2=CC=C(O)C(O)=C2)[C@H](O)C(O)[C@@H]1O | 3 |
| C17 | C_21_H_20_O_12_ | Hyperoside | OC[C@H]1O[C@@H](OC2=C(OC3=C(C(O)=CC(O)=C3)C2=O)C2=CC=C(O)C(O)=C2)[C@H](O)[C@@H](O)[C@H]1O | 3 |
| C18 | C_21_H_20_O_13_ | Gossypin | OC[C@H]1O[C@@H](OC2=C(O)C=C(O)C3=C2OC(=C(O)C3=O)C2=CC=C(O)C(O)=C2)[C@H](O)[C@@H](O)[C@@H]1O | 4 |
| C19 | C_27_H_28_O_19_ | Gossypetin-8-O-glucosyl-(1,2)-glucuronide | OC[C@H]1O[C@@H](OC2[C@@H](O)[C@H](O)C(O[C@H]2OC2=C(O)C=C(O)C3=C2OC(=C(O)C3=O)C2=CC=C(O)C(O)=C2)C(O)=O)C(O)C(O)[C@@H]1O | 4 |
| C20 | C_24_H_24_O_16_ | Gossypetin-8-O-ß-D-glucuronide glycerol ester | OCC(O)COC(=O)C1OC(OC2=C(O)C=C(O)C3=C2OC(=C(O)C3=O)C2=CC=C(O)C(O)=C2)C(O)C(O)C1O | 1 |
| C21 | C_28_H_30_O_19_ | Gossypetin 8-methylglucuronide 3-glucoside | COC(=O)C1O[C@@H](OC2=C(O)C=C(O)C3=C2OC(C2=CC=C(O)C(O)=C2)=C(O[C@@H]2O[C@H](CO)[C@@H](O)C(O)C2O)C3=O)C(O)[C@@H](O)[C@@H]1O | 3 |
| C22 | C_23_H_22_O_13_ | Hyperoside-3-acetate | CC(=O)OC1=CC(O)=CC2=C1C(=O)C(O[C@@H]1O[C@H](CO)[C@H](O)[C@H](O)[C@H]1O)=C(O2)C1=CC=C(O)C(O)=C1 | 3 |
| C23 | C_27_H_30_O_17_ | Quercetin-3-O-sophoroside | OC[C@H]1O[C@@H](O[C@@H]2[C@@H](O)[C@H](O)[C@@H](CO)O[C@H]2OC2=C(OC3=C(C(O)=CC(O)=C3)C2=O)C2=CC=C(O)C(O)=C2)[C@H](O)[C@@H](O)[C@@H]1O | 3 |
| C25 | C_21_H_18_O_14_ | Gossypetin-8-O-β-D-glucuronide | O[C@@H]1C(O)[C@H](OC2=C(O)C=C(O)C3=C2OC(=C(O)C3=O)C2=CC=C(O)C(O)=C2)OC([C@H]1O)C(O)=O | 4 |
| C26 | C_22_H_20_O_13_ | Carbonyl (quercetin-glucoside) | CO[C@H]1C(OC2=C(OC3=C(C(O)=CC(O)=C3)C2=O)C2=CC=C(O)C(O)=C2)[C@@H](O)[C@@H](O)O[C@@H]1C(O)=O | 4 |
| C27 | C_21_H_18_O_4_ | Isopongaflavone | COC1=CC2=C(C=CC(C)(C)O2)C2=C1C(=O)C=C(O2)C1=CC=CC=C1 | 21 |
| C28 | C_23_H_22_O_13_ | Hyperoside-7-acetate | CC(=O)OC1=CC2=C(C(O)=C1)C(=O)C(O[C@@H]1O[C@H](CO)[C@H](O)[C@H](O)[C@H]1O)=C(O2)C1=CC=C(O)C(O)=C1 | 3 |
| C29 | C_22_H_22_O_12_ | 3'-O-methyl-hyperoside | COC1=CC(=CC=C1O)C1=C(O[C@@H]2O[C@H](CO)[C@H](O)[C@H](O)[C@H]2O)C(=O)C2=C(O1)C=C(O)C=C2O | 3 |
| C30 | C_15_H_10_O_8_ | Gossypetin | OC1=CC=C(C=C1O)C1=C(O)C(=O)C2=C(O1)C(O)=C(O)C=C2O | 18 |
| C31 | C_23_H_22_O_13_ | Hyperoside-4'-acetate | CC(=O)OC1=CC=C(C=C1O)C1=C(O[C@@H]2O[C@H](CO)[C@H](O)[C@H](O)[C@H]2O)C(=O)C2=C(O1)C=C(O)C=C2O | 3 |
| C32 | C_27_H_24_O_14_ | Flavone glucoside | OC[C@H]1O[C@@H](OC2=C(OC3=C(C(O)=CC(O)=C3OC3=CC=C(O)C=C3)C2=O)C2=CC=C(O)C(O)=C2)C(O)C(O)[C@@H]1O | 3 |
| C33 | C_15_H_10_O_8_ | Myricetin | OC1=CC2=C(C(O)=C1)C(=O)C(O)=C(O2)C1=CC(O)=C(O)C(O)=C1 | 18 |
| C34 | C_23_H_22_O_14_ | Gossypetin 8-dimethylglucuronide | COC1C(O)C(O)C(OC2=C(O)C=C(O)C3=C2OC(=C(O)C3=O)C2=CC=C(O)C(O)=C2)OC1C(=O)OC | 4 |
| C35 | C_21_H_20_O_12_ | Isoquercitrin | OC[C@H]1O[C@@H](OC2=C(OC3=C(C(O)=CC(O)=C3)C2=O)C2=CC=C(O)C(O)=C2)[C@H](O)[C@@H](O)[C@@H]1O | 3 |
| C36 | C_27_H_24_O_14_ | Flavone glucoside | COC1=C(O)C2=C(OC(C3=CC=C(O)C(O)=C3)=C(O[C@@H]3O[C@H](CO)[C@@H](O)C(O)C3O)C2=O)C(OC2=C=CC=C2)=C1O | 3 |
| C37 | C_21_H_22_O_9_ | Isoliquiritin | OC[C@H]1O[C@@H](OC2=CC=C(\C=C\C(=O)C3=CC=C(O)C=C3O)C=C2)[C@H](O)[C@@H](O)[C@@H]1O | 1 |
| C38 | C_22_H_20_O_14_ | Gossypetin 8-O-methylglucuronide | COC(=O)C1O[C@@H](OC2=C(O)C=C(O)C3=C2OC(=C(O)C3=O)C2=CC=C(O)C(O)=C2)C(O)[C@@H](O)[C@@H]1O | 4 |
| C40 | C_23_H_22_O_14_ | Gossypetin 3-acetylglucoside | CC(=O)OC1C(CO)OC(OC2=C(OC3=C(C(O)=CC(O)=C3O)C2=O)C2=CC=C(O)C(O)=C2)C(O)C1O | 3 |
| C41 | C_22_H_20_O_13_ | Carbonyl (quercetin-glucoside) | OCC1OC(OC2=C(OC3=C(C(O)=C(C=O)C(O)=C3)C2=O)C2=CC=C(O)C(O)=C2)C(O)C(O)C1O | 3 |
| C42 | C_23_H_22_O_13_ | Isoquercitrin-3''-acetate | CC(=O)OC1[C@H](O)C(CO)O[C@@H](OC2=C(OC3=C(C(O)=CC(O)=C3)C2=O)C2=CC=C(O)C(O)=C2)[C@@H]1O | 3 |
| C43 | C_15_H_10_O_7_ | Quercetin | OC1=CC2=C(C(O)=C1)C(=O)C(O)=C(O2)C1=CC=C(O)C(O)=C1 | 18 |
| C44 | C_27_H_24_O_14_ | Flavone glucoside | OC[C@H]1O[C@@H](OC2=C(OC3=C(C(O)=CC(O)=C3C3=CC=C(O)C(O)=C3)C2=O)C2=CC=C(O)C(O)=C2)C(O)C(O)[C@@H]1O | 3 |
| C45 | C_23_H_22_O_14_ | 4'-methylgossypetin 8-methylglucuronide | COC(=O)C1OC(OC2=C(O)C=C(O)C3=C2OC(=C(O)C3=O)C2=CC=C(OC)C(O)=C2)C(O)C(O)C1O | 4 |
| C49 | C_27_H_24_O_14_ | Flavone glucoside | OC[C@@H]1CC(O)C(O)[C@H](OC2=C(OC3=C(C(O)*=CC(OC4=CC=C(O)C(O)=C4)=C3)C2=O)C2=CC=C(O)C(O)=C2)O1 | 3 |

# Table S6 40 potential targets directly involved in HK against CKD.

| No. | Target | Common Name | Uniprot ID | Target Class | Degree |
| --- | --- | --- | --- | --- | --- |
| 1 | Aldose reductase | AKR1B1 | P07943 | Voltage-gated ion channel | 40 |
| 2 | Cyclooxygenase-2 | PTGS2 | P35355 | Family A G protein-coupled receptor | 40 |
| 3 | Carbonic anhydrase II | CA2 | P27139 | Transferase | 38 |
| 4 | Arachidonate 5-lipoxygenase | ALOX5 | P12527 | Transferase | 13 |
| 5 | Acetylcholinesterase | ACHE | P37136 | Nuclear receptor | 4 |
| 6 | Cytochrome P450 1A1 | CYP1A1 | P00185 | Cytochrome P450 | 4 |
| 7 | 5'-nucleotidase | NT5E | P21588 | Enzyme | 3 |
| 8 | Amyloid beta A4 protein | APP | P08592 | Secreted protein | 3 |
| 9 | Androgen Receptor | AR | P15207 | Enzyme | 3 |
| 10 | Arachidonate 12-lipoxygenase | ALOX15 | Q02759 | Protease | 3 |
| 11 | Arginase-1 | ARG1 | P07824 | Protease | 3 |
| 12 | Beta-glucuronidase | GUSB | P06760 | Enzyme | 3 |
| 13 | DNA topoisomerase 1 | TOP1 | Q9WUL0 | Enzyme | 3 |
| 14 | Glycogen synthase kinase-3 beta | GSK3B | P18266 | Protease | 3 |
| 15 | Liver glycogen phosphorylase | PYGL | P09811 | Enzyme | 3 |
| 16 | Plasminogen | PLG | Q01177 | Enzyme | 3 |
| 17 | Poly [ADP-ribose] polymerase 1 | PARP1 | P27008 | Lyase | 3 |
| 18 | Vasopressin V2 receptor | AVPR2 | Q00788 | Electrochemical transporter | 3 |
| 19 | Coagulation factor X | F10 | Q63207 | Ligand-gated ion channel | 2 |
| 20 | Acid ceramidase | ASAH1 | Q6P7S1 | Voltage-gated ion channel | 1 |
| 21 | Aryl hydrocarbon receptor | AHR | P41738 | Ligand-gated ion channel | 1 |
| 22 | Bradykinin B1 receptor | BDKRB1 | P97583 | Enzyme | 1 |
| 23 | cAMP and cAMP-inhibited cGMP 3',5'-cyclic phosphodiesterase 10A | PDE10A | Q9QYJ6 | Enzyme | 1 |
| 24 | Cannabinoid CB1 receptor | CNR1 | P20272 | Other cytosolic protein | 1 |
| 25 | Carnitine palmitoyltransferase 1A | CPT1A | P32198 | Family A G protein-coupled receptor | 1 |
| 26 | Cathepsin K | CTSK | O35186 | Protease | 1 |
| 27 | Cathepsin L | CTSL | P07154 | Enzyme | 1 |
| 28 | C-C chemokine receptor type 1 | CCR1 | Q9JLY8 | Electrochemical transporter | 1 |
| 29 | Cytochrome P450 17A1 | CYP17A1 | P11715 | Enzyme | 1 |
| 30 | Glutamate NMDA receptor; Grin2b | GRIN2B | Q00960 | Ligand-gated ion channel | 1 |
| 31 | Histone deacetylase 1 | HDAC1 | Q4QQW4 | Nuclear receptor | 1 |
| 32 | Monoamine oxidase A | MAOA | P21396 | Family A G protein-coupled receptor | 1 |
| 33 | Neurokinin 3 receptor | TACR3 | P16177 | Enzyme | 1 |
| 34 | P2X purinoceptor 7 | P2RX7 | Q64663 | Kinase | 1 |
| 35 | Prostanoid EP4 receptor | PTGER4 | P43114 | Family A G protein-coupled receptor | 1 |
| 36 | Serotonin 2a (5-HT2a) receptor | HTR2A | P14842 | Kinase | 1 |
| 37 | Sodium/glucose cotransporter 1 | SLC5A1 | P53790 | Electrochemical transporter | 1 |
| 38 | Sodium/glucose cotransporter 2 | SLC5A2 | P53792 | Electrochemical transporter | 1 |
| 39 | Testis-specific androgen-binding protein | SHBG | P08689 | Family A G protein-coupled receptor | 1 |
| 40 | Tryptophan 2,3-dioxygenase | TDO2 | P21643 | Voltage-gated ion channel | 1 |
